# Translation Initiation Factors eIF3 and HCR1 Control Translation Termination and Stop Codon Read-Through in Yeast Cells

**DOI:** 10.1371/journal.pgen.1003962

**Published:** 2013-11-21

**Authors:** Petra Beznosková, Lucie Cuchalová, Susan Wagner, Christopher J. Shoemaker, Stanislava Gunišová, Tobias von der Haar, Leoš Shivaya Valášek

**Affiliations:** 1 Laboratory of Regulation of Gene Expression, Institute of Microbiology ASCR, Videnska, Prague, the Czech Republic; 2 Howard Hughes Medical Institute, Johns Hopkins University School of Medicine, Baltimore, Maryland, United States of America; 3 School of Biosciences, University of Kent, Kent, United Kingdom; National Institute of Child Health and Human Development, NIH, United States of America

## Abstract

Translation is divided into initiation, elongation, termination and ribosome recycling. Earlier work implicated several eukaryotic initiation factors (eIFs) in ribosomal recycling *in vitro*. Here, we uncover roles for HCR1 and eIF3 in translation termination *in vivo*. A substantial proportion of eIF3, HCR1 and eukaryotic release factor 3 (eRF3) but not eIF5 (a well-defined “initiation-specific” binding partner of eIF3) specifically co-sediments with 80S couples isolated from RNase-treated heavy polysomes in an eRF1-dependent manner, indicating the presence of eIF3 and HCR1 on terminating ribosomes. eIF3 and HCR1 also occur in ribosome- and RNA-free complexes with both eRFs and the recycling factor ABCE1/RLI1. Several eIF3 mutations reduce rates of stop codon read-through and genetically interact with mutant eRFs. In contrast, a slow growing deletion of *hcr1* increases read-through and accumulates eRF3 in heavy polysomes in a manner suppressible by overexpressed ABCE1/RLI1. Based on these and other findings we propose that upon stop codon recognition, HCR1 promotes eRF3·GDP ejection from the post-termination complexes to allow binding of its interacting partner ABCE1/RLI1. Furthermore, the fact that high dosage of ABCE1/RLI1 fully suppresses the slow growth phenotype of *hcr1Δ* as well as its termination but not initiation defects implies that the termination function of HCR1 is more critical for optimal proliferation than its function in translation initiation. Based on these and other observations we suggest that the assignment of HCR1 as a *bona fide* eIF3 subunit should be reconsidered. Together our work characterizes novel roles of eIF3 and HCR1 in stop codon recognition, defining a communication bridge between the initiation and termination/recycling phases of translation.

## Author Summary

Protein synthesis (translation) utilizes genetic information carriers, mRNAs, as templates for the production of proteins of various cellular functions. Typically it is divided into four phases: initiation, elongation, termination and ribosomal recycling. In this article we argue that the strict mechanistic separation of translation into its individual phases should be reconsidered in the light of “multitasking” of initiation factors eIF3, HCR1 and ABCE1/RLI1. In detail, we show that eIF3 and HCR1 not only promote the initiation phase but also specifically act at the other end of the translational cycle during termination. We present genetic and biochemical data linking eIF3 and HCR1 with both eukaryotic release factors (eRF1 and eRF3) and the ribosomal recycling factor ABCE1/RLI1, and propose a model for how all these factors co-operate with each other to ensure stringent selection of the stop codon. Collectively, our findings suggest that changes in one phase of translation are promptly communicated to and coordinated with changes in the other phases to maintain cellular homeostasis of all ongoing processes.

## Introduction

Protein synthesis or mRNA translation is a complex and highly conserved process that can be separated into initiation, elongation, termination and ribosome recycling phases. Although these four phases are distinct in time, there is a longstanding notion for some form of communication among them. Notably, several initiation factors and related proteins have been proposed to function in more than one phase. These include ABCE1/RLI1 and GLE1, which are believed to promote both the initiation and termination phases by a mechanism that remains to be elucidated [1]–[3], eIF5A proposed to stimulate all three major phases [4], and the *bona fide* translation initiation factor eIF3, which has been recently suggested to promote the recycling phase, at least in a mammalian *in vitro* reconstituted system [5], [6].

The beginning of a translational cycle involves a series of steps that culminate in the assembly of the 80S initiation complex (IC) on the AUG start codon (reviewed in [7]). These steps include 1) Met-tRNA_i_^Met^ recruitment to the 40S subunit to form the 43S pre-initiation complex (PIC), 2) mRNA recruitment to the 43S PIC to form the 48S PIC, 3) scanning of the 48S PIC to the first recognized start codon, and 4) joining of the 60 subunit to commit the resulting 80S IC to the elongation phase. The translation initiation factor eIF3, which in yeast consists of five essential core subunits (eIF3a/TIF32, b/PRT1, c/NIP1, i/TIF34 and g/TIF35) and one transiently associated, non-essential subunit (eIF3j/HCR1), is actively involved in regulation of the first three of these steps [7]. In the PIC assembly steps, the action of eIF3 is further stimulated by one of its interacting partners, the ATP-binding cassette protein ABCE1/RLI1, by an unknown mechanism [1]. In contrast to the most of eIFs, eIF3 interacts with the solvent-exposed side of the small ribosomal subunit [7] and as such it was proposed to be able to interact with active 80S ribosomes post-initiation [8]–[10].

The end of a translational cycle involves another series of steps that culminate in the release of a newly synthesized polypeptide from the translating ribosome (the termination phase), and in the dissolution of the ribosome:tRNA:mRNA complex (the recycling phase). Termination begins when a stop codon enters the ribosomal A-site, forming a pre-termination complex (pre-TC) [11]. In eukaryotes, all three stop codons are decoded by the eukaryotic release factor 1 (eRF1). According to recent models [12], [13], eRF1 enters the ribosomal A-site in complex with a second release factor, eRF3, in its GTP bound form. Recognition of a stop codon triggers GTPase activity of eRF3, which leads to its dissociation from the complex in its GDP bound form. eRF1 is then free to activate the ribosomal peptidyl transferase centre (PTC), which hydrolyses the bond between the P-site tRNA and the nascent polypeptide. Importantly, these steps are promoted by RLI1 in an ATP-independent manner; i.e. by the same factor that also somehow stimulates the eIF3 function in the initiation phase. Molecular details of this RLI1 role in termination are similarly not known, nevertheless, the proposed active role of RLI1 in stop codon recognition is consistent with observations that conditional down regulation of RLI1 protein levels increases stop codon read-through in yeast [2]. Based on the most recent structural model, RLI1 binds to the same site on the terminating ribosome as eRF3 (thus their binding is mutually exclusive), and its 4Fe-4S domain interacts with the C-terminal domain of eRF1 to push the conserved GGQ motif in the middle domain of eRF1 to the PTC next to the acceptor stem of the P-site tRNA to trigger polypeptide release [13].

Recycling of eRF1-associated post-termination complexes (post-TCs) is also mediated by ABCE1/RLI1, this time, however, in an ATP-dependent manner [6], [12]. It was hypothesized that RLI1, upon binding and hydrolyzing ATP, switches its conformation into a closed state, and the mechanochemical work generated by this switch splits post-TCs into free 60S subunits and deacylated tRNA- and mRNA-bound 40S subunits (40S-post-TC) [13]. Finally, Pisarev *et al.* showed that the release of tRNA and mRNA from the 40S-post-TCs is *in vitro* ensured by the *bona fide* initiation factors eIF1, eIF1A and eIF3 [5], [6]. eIF3, and in particular its j subunit (HCR1 in yeast), were suggested to play the key role in mRNA dissociation.

Since the implication of eIF3 in the recycling process was deduced only from experiments carried out with 11-codon long model mRNAs in mammalian *in vitro* reconstituted systems, we decided to investigate whether or not eIF3 also plays a direct role in translation termination and/or ribosomal recycling in the living cell. Here we show that the five core eIF3 subunits and HCR1 control translation termination and stop codon read-through in yeast, although in the opposite manner. HCR1 specifically cooperates with eRF3 and RLI1 and based on our and previous findings we propose that HCR1 and its mammalian orthologue should no longer be considered as *bona fide* subunits of eIF3. In any case, involvement of the canonical translation initiation factor eIF3 in termination strongly supports the idea that there is a highly coordinated communication between individual translational phases.

## Results

### Mutations in eIF3 subunits and hcr1 deletion affect stop codon read-through

eIF3 and the eIF3-core-associated factors like HCR1 and RLI1 play a role in ribosome recycling – at least *in vitro* [5], [6], while only RLI1 is to date known to somehow promote also the preceding translation termination step [2]. In order to address whether eIF3 itself is likewise functionally involved in translation termination, we first measured the frequency of stop codon read-through in a collection of eIF3 mutants using an established dual-luciferase reporter assay, specifically designed to be independent of mRNA levels [14]. This reporter system is similar to the one which was also used to demonstrate increased stop codon read-through upon conditional down-regulation of RLI1 [2]. The [*psi^−^*] strain background used in these initial experiments contains a genome-encoded UGA suppressor tRNA leading to unusually high basal UGA read-through levels of 3–4%, which is however ideal for studying stop-codon read-through effects. Importantly, as shown below, the results we obtained are independent of the presence of this suppressor tRNA.

**Figure 1:**
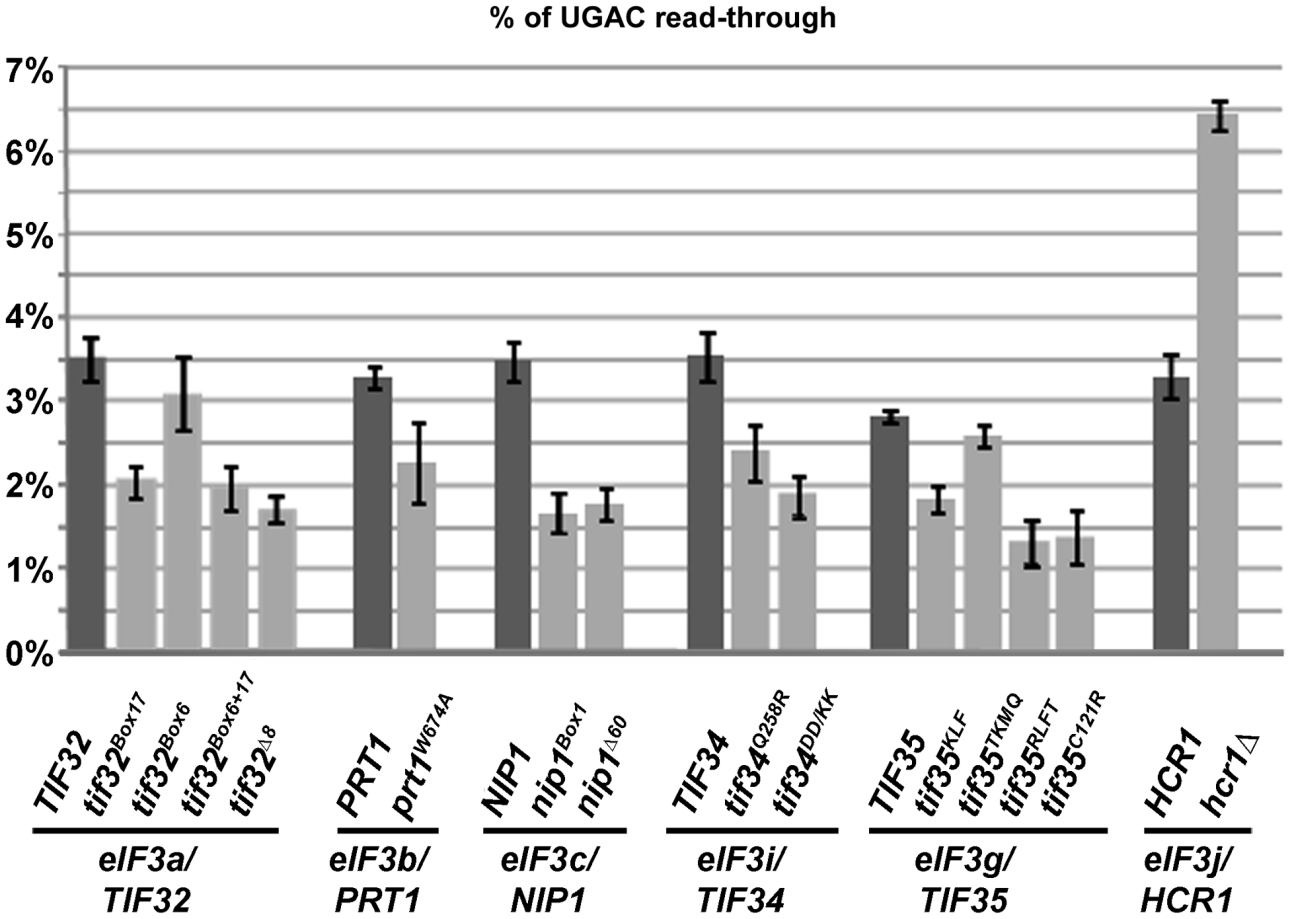
Mutations reducing the activity of translation initiation factor eIF3 and HCR1 affect stop codon read-through. Stop codon read-through was measured using dual luciferase reporter constructs as described in the main text. Plasmid-born mutant alleles of genes encoding eIF3 subunits and HCR1 were introduced into their respective shuffling strains, which are derived from a common strain background (for details please see Text S1 and Table S1). The wt strain background has unusually high levels of UGA read-through, due to the presence of an opal (UGA) suppressor tRNA in the genome. For each independently derived shuffling strain, read-through is shown for pairs of strains shuffled with wt or the indicated mutant alleles of the gene in question. For the non-essential HCR1 subunit, the H3675 *hcr1Δ* strain is shown. All investigated mutants showed significant (p<0.05) reductions in the level of stop codon read-through, with the exception of *tif35^TKMQ^*, which showed no significant difference, and *Δhcr1* which showed strong and significant increase in stop codon read-through.

The eIF3 mutants that were chosen for read-through analysis were previously shown to affect multiple initiation steps, from the 43 PIC assembly (due to reduced 40S-binding affinity of eIF3) to scanning for AUG recognition (with wild-type 40S-binding affinity of eIF3); summarized in Table S1. Strikingly, the majority of mutations in the core eIF3 subunits that we tested showed a significant reduction in stop-codon read-through (Figure 1) that thus could not be simply attributed to the reduced eIF3 association with ribosomes. Also, since the effect of eIF3 mutations on translation initiation does not correlate well with the observed translation termination defect (such as, for example, in case of *prt1-W674A vs. tif34-DD/KK* or *tif35-TKMQ vs. tif35-RLFT* mutants), we conclude that the impact of these mutations on translation initiation vs. termination is genetically separable. Importantly, in contrast to all core eIF3 subunits, deletion of the non-essential *hcr1* gene encoding eIF3j resulted in significantly increased stop-codon read-through (Figure 1), similar to that reported for RLI1 down-regulation [2]. Neither eIF3 mutations nor *hcr1Δ* have any impact on eRF1, eRF3 and RLI1 protein levels.

In order to confirm this unexpected result and to explore whether the observed effect on translation termination was specific to eIF3 or common to all other members of the Multifactor complex (MFC; composed of eIF1, eIF2, eIF3 and eIF5) and their closely co-operating factor eIF1A, we used partial depletion alleles (DaMP alleles) for these essential factors from the genome-wide DaMP collection [15]. DaMP alleles contain a selectable marker cassette inserted into the 3′-UTR of a gene, leading to destabilization of the respective mRNA *via* the nonsense mediated decay pathway (NMD).

By their nature, DaMP alleles show varying degrees of depletion for different genes, and data obtained with DaMP alleles have to be interpreted with this in mind. Since the depleted genes are all essential, loss of the corresponding gene product below a critical level will affect growth, and demonstration of reduced growth for an individual strain can thus be taken as a reliable indicator for depletion below a critical threshold. In contrast, an absence of growth phenotypes cannot be unambiguously interpreted, as depletion of the gene product may have occurred but may have remained above a level where fitness is detectably affected. It should be noted that growth phenotypes are a better indicator of functional depletion than assessment of physical expression levels by Western blotting, since the relationship between translation factor abundance and translation rates is non-linear and generally not predictable [16]. Finally, since the DaMP strain background does not contain a UGA suppressor tRNA in the genome, these data also exclude possible suppressor tRNA effects on the eIF3 mutants in the initial experiments.

When we compared growth rates of the MFC DaMP alleles to the corresponding wild type (wt) strain (Figure S1), we observed that most non-eIF3 MFC factors but only one of the eIF3 strains (*TIF35*) showed a growth phenotype indicative of a significant depletion. When we proceeded to assess stop codon read-through in these strains, we observed that the one eIF3 strain for which the growth assay indicated significant depletion (about 2.5-fold as determined by Western blotting) also showed significantly reduced stop codon read-through. In addition, the *NIP1* depletion strain also showed modest but significant reduction in stop-codon read-through, which may be caused by depletion of the gene product to a level that does not yet affect growth rates. In contrast, none of the depletion alleles for the non-eIF3 MFC component showed a reduction in read-through, although eIF1A and eIF2α showed small but significant increases in stop codon read-through and, interestingly, eIF2γ showed even higher increase (∼2-fold) that is similar to *hcr1Δ*. While the mechanism behind the increased read-through in the eIF1A and eIF2 alleles is yet to be explored, these observations demonstrate that i) reductions in eIF3 activity reliably lead to reductions in stop-codon read-through levels, whether this reduction is caused by point mutations or other gene ablation alleles, ii) this effect is specific to core eIF3 subunits, whereas other MFC components and HCR1 display either none or the opposite phenotype, and iii) general reduction in the initiation rates does not automatically affect the precision of translation termination – see for example the read-through data for eIF1 (Figure S1), the protein levels of which were depleted by ∼5-fold – suggesting that the reduced levels of read-through displayed by eIF3 mutants do not necessarily occur as an indirect consequence of an overall compromised protein synthesis. Interestingly, a similar phenotype (reduced read-through) was also observed for overexpression of eRF1 in otherwise wt cells (Figure S2; combined overexpression of eRF1 and 3 led to further, modest exacerbation), indicating that the eIF3 mutants may somehow affect the availability of eRFs for the stop codon in the A-site.

### Overexpression of ABCE1/RLI1 fully suppresses the growth and read-through but not the initiation defects of the *hcr1* deletion strain

Next we wished to explore a molecular mechanism of the increased stop-codon read-through phenotype displayed by deletion of the non-essential *hcr1* gene (Figure 1), which sharply contrasted with the opposite termination phenotype of mutant core eIF3 subunits (Figure 1). As discussed above, recent reports suggested that the ABCE1/RLI1 protein not only critically promotes ribosomal recycling [6], [12], [17] but is also somehow involved in translation termination, as its conditional depletion produced an increased read-through defect [2]. Moreover, RLI1 was also implicated in biogenesis and transport of pre-ribosomes from the nucleolus [18] and in stimulating translation initiation by promoting assembly of 43S PICs together with eIF3 [1]. The striking resemblance of the latter effects with the previously reported functions of HCR1 [18]–[24] plus the earlier observations that RLI1 directly interacts with HCR1 *via* its ABC2 domain [2] and that combination of *hcr1Δ* with the TAP-tagged RLI1 results in synthetic lethality [18] prompted us to test a potential functional redundancy between these two proteins.

**Figure 2:**
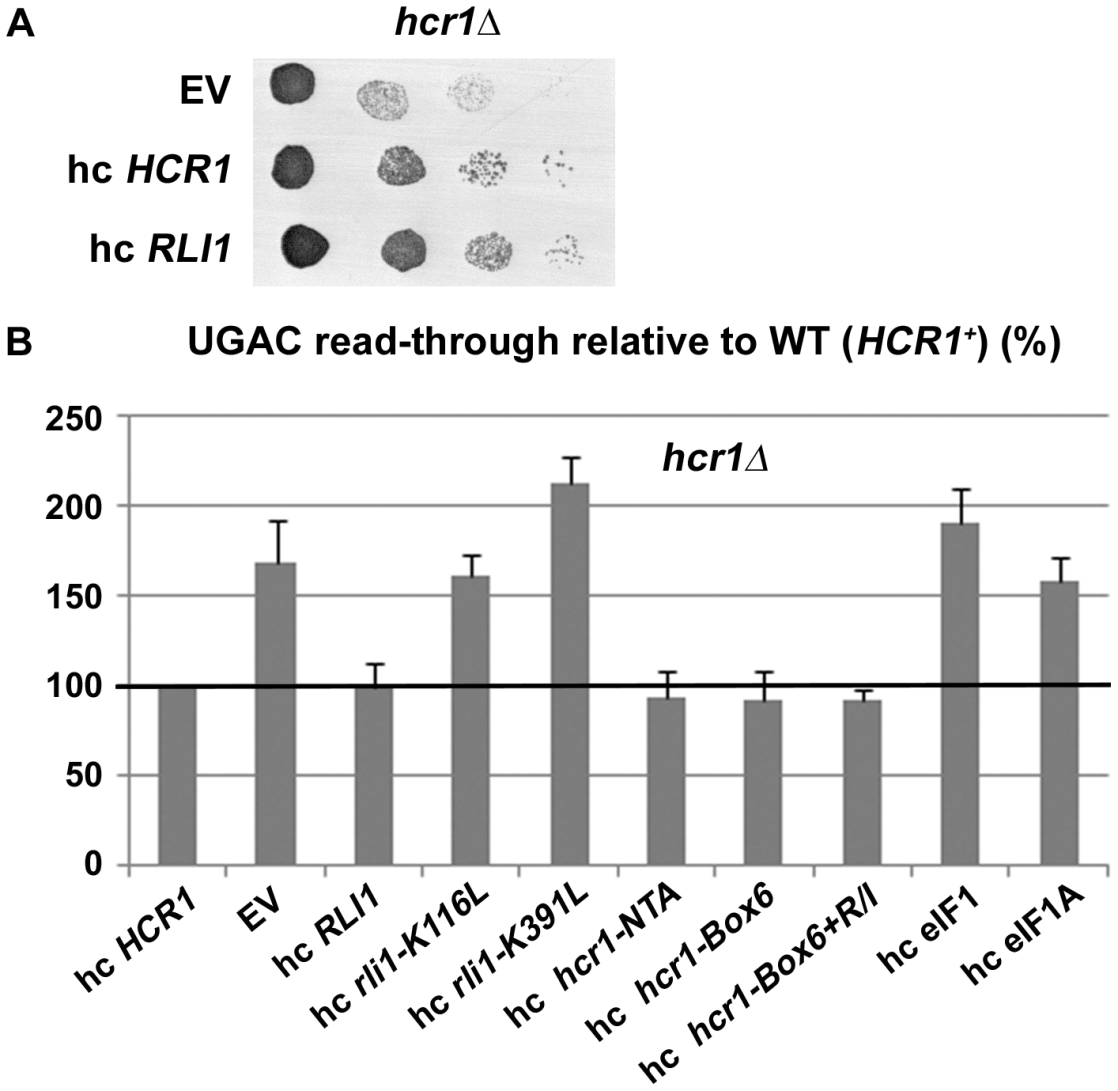
Increased gene dosage of ABCE/RLI1 suppresses the slow growth and read-through defects of *hcr1Δ*. (**A**) The *hcr1Δ* strain was transformed with either empty vector (EV), hc *HCR1* or hc *RLI1*. The resulting transformants were subjected to a growth spot assay at 30°C for 2 days. (**B**) The *hcr1Δ* strain was transformed with hc vectors carrying either wt or mutant *HCR1* and *RLI1* alleles, and *SUI1* (eIF1) and *TIF11* (eIF1A). The resulting transformants were grown in SD and analyzed for stop codon read-through as described in Figure 1. Thus obtained values were normalized to the value obtained with the *hcr1Δ* strain transformed with wt *HCR1*, which was set to 100%.

Strikingly, we found that overexpression of RLI1 (about 2.5-fold) fully suppressed the slow growth defect of an *hcr1Δ* strain (Figure 2A). Moreover, high copy (hc) *RLI1* also fully suppressed the increased read-through phenotype of this strain (Figure 2B). By way of control, we overexpressed elongation factor eEF3 (encoded by *YEF3*) as an independent ABC cassette-containing protein engaged in translation, which had no effect on the growth or read-through phenotypes of the *hcr1Δ* strain (Figure S3A and B). In addition, hc RLI1 did not suppress the increased read-through defect of the DaMP eIF2γ mutant (data not shown), further underscoring the novelty of the proposed role for HCR1 and eIF3 in termination.

Importantly, no hc suppression was observed when either the formation of the RLI1 N-terminal 4Fe-4S clusters (C25S and C61S mutants) or the ATP binding by its ABC cassettes (K116L, K391L, G224D G225D and G470D G471D mutants) were compromised (Figure 2B and data not shown). The integrity of the crucial N-terminal region of RLI1 as well as its intact ATPase activity are therefore critically required for a functional replacement of HCR1. In the opposite arrangement, hc *HCR1* suppressed neither the slow growth nor the increased read-through phenotype of the *Tet::RLI1* conditional depletion strain (Figure S4). It is noteworthy that in agreement with earlier results [2], [12], [17], the intact N-terminal 4Fe-4S clusters and the ability of RLI1 to bind and hydrolyze ATP were absolutely essential for restoration of the read-through defect in the *Tet::RLI1* cells (Figure S4).

In order to find out if HCR1 acts independently of eIF3 in the termination process, we examined the read-through phenotype of *hcr1* mutations, which are known to eliminate binding of full length HCR1 to eIF3 [21], [22]. As shown in Figure 2B (mutations *hcr1-Box-NTA, -Box6* and -*Box6+R/I*), no effect was observed implying that the HCR1 function in termination does not require its physical association with eIF3.

One of the major initiation phenotypes of *hcr1Δ* is a leaky scanning defect (a decreased ability to recognize AUG as the translational start site resulting in increased scanning past it), which can be suppressed by hc eIF1A [21]. As can be seen in Figure 2B, neither hc eIF1A nor eIF1 suppressed the read-though defect of *hcr1Δ*. Similarly, hc *RLI1* did not suppress the leaky scanning defect of *hcr1Δ* (Figure S5). Hence, these findings clearly suggest that the *hcr1Δ* defects in initiation and termination are genetically separable and that RLI1 cannot replace HCR1 in all of its functions. Importantly, however, since hc eIF1A suppressed the *hcr1Δ* growth defect only partially [21], as opposed to the full suppression by hc *RLI1* (Figure 2A), we propose that the major contributor to the *hcr1Δ* slow growth phenotype is not a defect in initiation, as previously believed, but a defect related to translation termination. Altogether it seems that this substoichiometric “subunit” of eIF3 works more independently of the core eIF3 than previously thought and therefore we suggest not considering HCR1 as a *bona fide* eIF3 subunit anymore (see Discussion for more details).

### Complexes containing eIF3, HCR1, ABCE1/RLI1 and both eRFs, free of ribosomes and RNA, occur i*n vivo*

**Figure 3:**
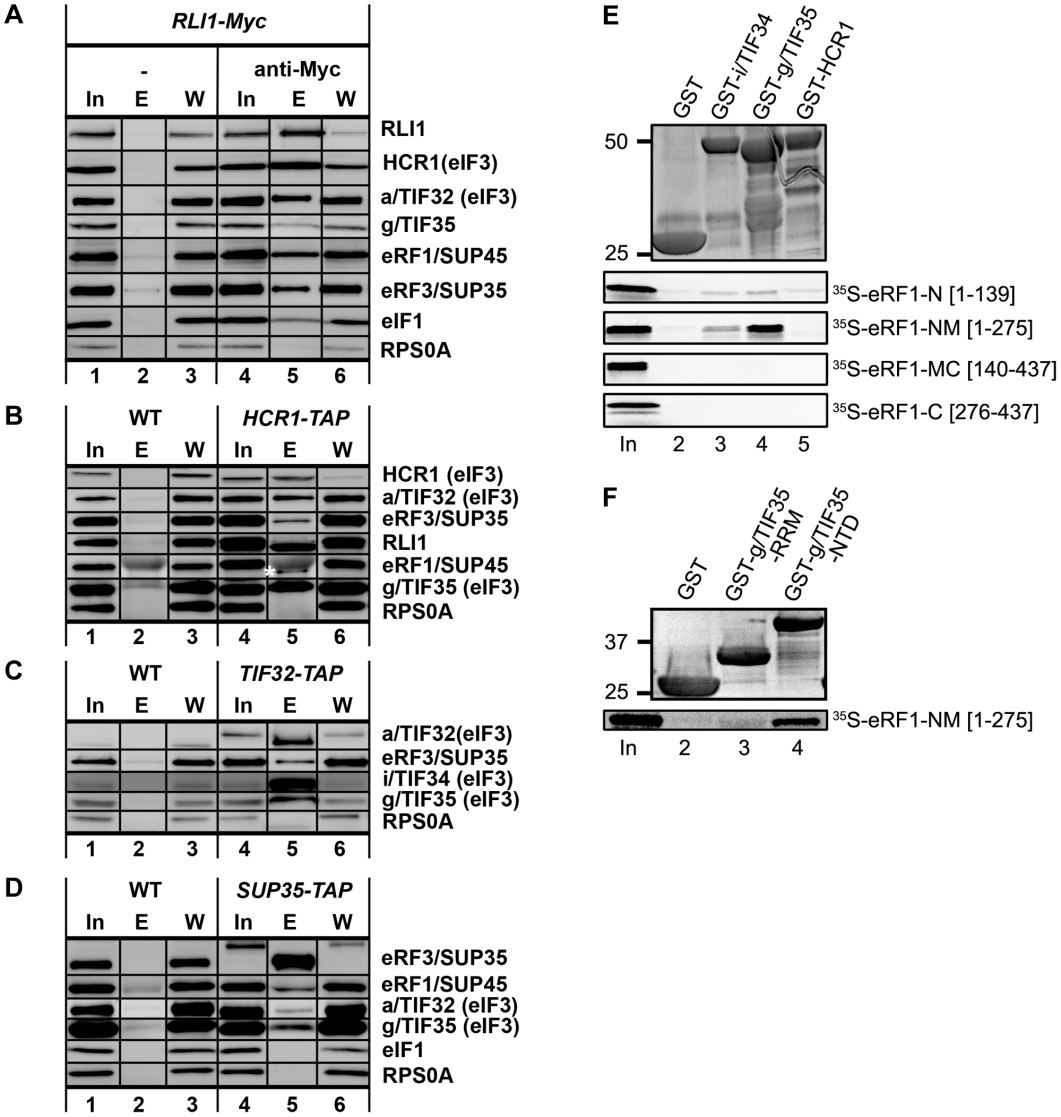
Complexes containing eIF3, HCR1, ABCE1/RLI1 and both eRFs, free of ribosomes and RNA, occur *in vivo*; and the NTD of g/TIF35 and i/TIF34 directly interact with the N and M domains of eRF1. (**A**) WCEs were prepared from YDH353 bearing chromosomal Myc-tagged *RLI1* and immunoprecipitated with or without anti-Myc antibodies. The immune complexes were subjected to Western analysis. In, 5% of input; E, 100% of the elution fraction; W, 5% of the supernatant fraction. Also note that anti-RLI1 and -eRF1 antibodies were raised for the purpose of this study. (**B**) WCEs were prepared from HCHO-treated (1%) cells bearing wt (H2879) or TAP-tagged (H553) chromosomal alleles of *HCR1* and incubated with IgG Sepharose 6 Fast Flow beads. The immune complexes were eluted by boiling in the SDS buffer and subjected to Western analysis. In, 1.5% of input; E, 50% of the elution fraction; W, 1.5% of the supernatant fraction. eRF1 is indicated by an asterisk below the immunoglobulins. (**C**) WCEs from HCHO-treated cells (1%) cells bearing wt (H2879) or TAP-tagged (H555) chromosomal alleles of *TIF32* were processed as in panel B except that the immune complexes were eluted by TEV protease cleavage. In, 1.5% of input; E, 100% of the elution fraction; W, 1.5% of the supernatant fraction. (**D**) WCEs from HCHO-treated cells (1%) cells bearing wt (74D-694) or TAP-tagged (H517) chromosomal alleles of *SUP35* were processed as in panel C. (**E**) Full-length i/TIF34 (lane 3), g/TIF35 (lane 4), and HCR1 (lane 5) fused to GST, and GST alone (lane 2), were tested for binding to ^35^S-labeled individual domains of eRF1; 10% of input amounts added to each reaction is shown in lane 1 (In). (**F**) The RRM (lane 3) and N-terminal (lane 4) domains of g/TIF35 fused to GST, and GST alone (lane 2), were tested for binding to ^35^S-labeled NM domains of eRF1; 10% of input amounts added to each reaction is shown in lane 1.

If eIF3 and HCR1 are indeed involved in translation termination as our read-through data indicate (Figure 1), it should be possible to detect a complex between these molecules and the release factors *in vivo*. We therefore carried out a series of *in vivo* pull down experiments using Myc-tagged RLI1, or TAP-tagged HCR1, a/TIF32 or eRF3 as baits. To stabilize presumably only transient interactions between eIF3 and termination/recycling factors, the TAP-tag experiments were performed after modest (1%) pre-treatment of growing cells with formaldehyde as described in [25]. As shown in Figure 3A, Myc-tagged RLI1 specifically pulled down selected eIF3 subunits (∼51±1% of the input for a/TIF32 and ∼43±2.6% for g/TIF35) and HCR1, as shown before [1]. In addition and in contrast to the latter study, we also observed significant co-precipitation of both release factors (∼22±1.7% for eRF1 and ∼13±3.9% for eRF3). The TAP-tagged HCR1 repeatedly co-purified with selected eIF3 subunits, as expected, but also with RLI1 (∼58±5.8%) and small but specific amounts of eRF3 (9±0.2%) and eRF1 (∼2±0.2%) (Figure 3B; eRF1 is indicated by an asterisk). eRF3 also co-precipitated with TAP-tagged a/TIF32 (∼16±4.7%), and, importantly, TAP-tagged eRF3 reproducibly and specifically brought down small but significant amounts of core eIF3 subunits (∼6.4±0.7% for a/TIF32 and ∼18±5.8% for g/TIF35) but no other MFC-members such as eIF1 (Figure 3C and D; note that the mobility of a/TIF32 and eRF3 vary between Input and Elution lanes due to a TEV protease-mediated cleavage of the TAP tag). We also tested the TAP-tagged eRF1 strain, however, no proteins were recovered – not even the TAP-eRF1 by itself – indicating that this particular fusion allele is not functional. Importantly, the yield of neither of these experiments was affected by RNase A treatment (Figure S6) and no ribosomes were present in the purified complexes (see RPS0A strips in panels A–D) strongly suggesting that the ribosome- and RNA-free complexes of eIF3, HCR1, eRF1, eRF3 and RLI1 do exist in the cytoplasm. More specifically, these experiments show that eIF3 and HCR1 contacts all critical termination players discussed in this study, though we cannot conclude whether all these factors occur in one single super-complex, or whether we are pulling down their partial subcomplexes.

### The N-M domains of eRF1 directly interact with the N-terminal domain of g/TIF35 and weakly also with i/TIF34 *in vitro*

The fact that eIF3 and HCR1 associate with eRFs *in vivo* and that their mutations affect fidelity of the termination process prompted us to test protein-protein interactions between eIF3 subunits, HCR1 and both eRFs. We fused individual eIF3 subunits and HCR1 to a GST moiety and used these fusions in pull-down assays with *in vitro* synthesized, radiolabeled, well-defined domains of eRF1 and eRF3. As shown in Figure 3E, the N-terminal and Middle (N-M) domains but not the middle and C-terminal (M-C) domains of eRF1 specifically interacted with GST-g/TIF35 and weakly also with GST-i/TIF34, in contrast to GST-HCR1 and a negative control of the GST protein alone. (The N domain of eRF1 carries determinants of the stop codon recognition; the M domain contains the conserved GGQ motif required for peptide release; and the C domain interacts with eRF3.) Interestingly, DOM34/Pelota, the release-like factor closely related in sequence and structure to eRF1, also binds eIF3g in human cell lines [26], albeit in this case *via* Pelota's C-terminal domain. No interactions between eIF3 subunits and eRF3 were observed. g/TIF35 can be divided into the N-terminal Zn-finger and C-terminal RRM domains and our GST pull down experiments revealed that eRF1-NM binds specifically to the N-terminal Zn-finger domain of g/TIF35 (Figure 3F). Hence we propose that eIF3 and eRF1 are in a direct contact *via* two small eIF3 subunits and the NTD of eRF1, which requires further support from the M domain to get fully engaged in these interactions.

### eIF3 and HCR1 associate with 80S couples isolated from heavy polysomes

**Figure 4:**
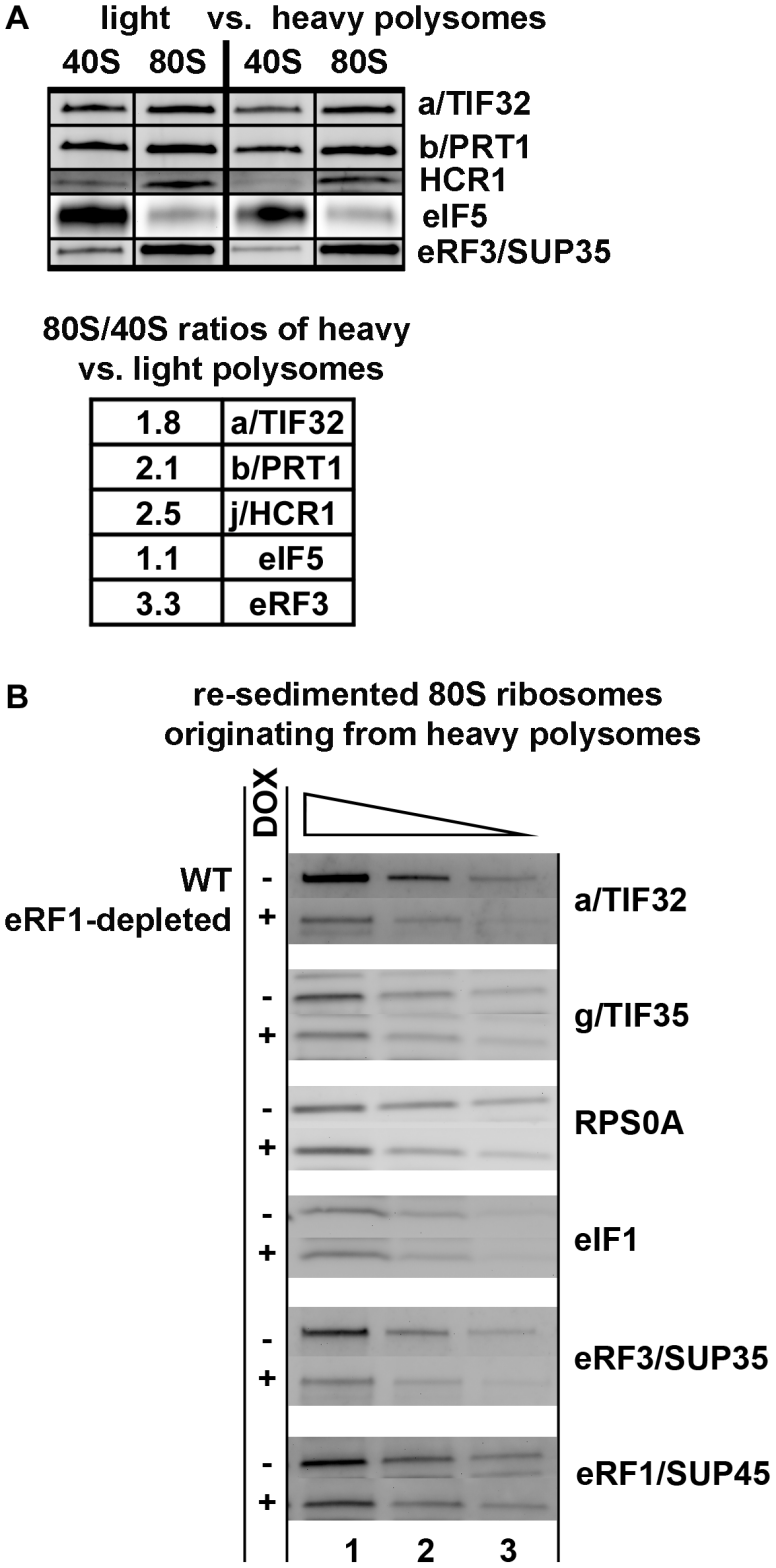
eIF3 associates with 80S couples isolated from heavy polysomes in an eRF1-dependent manner. (**A**) The wt strain (H2819) was grown in SD medium at 30°C to an OD_600_ of ∼1 and cross-linked with 0.5% HCHO prior to harvesting. WCEs were prepared, separated on a 5%–45% sucrose gradient by centrifugation at 39,000 rpm for 2.5 h and two collected fractions containing either disomes and trisomes or pentasomes and heavier polysomes were treated with RNase A to separate the initiating PICs from 80S couples on mRNAs and subjected to the sucrose gradient resedimentation protocol [25]. Two fractions containing 43-48S PICs and 80S ribosomes from each polysomal pool were collected and subjected to Western blot analysis; the ratio of the 80S/40S ratios for heavy over light polysomes was calculated and plotted for each factor. This experiment was repeated four times. (**B**) eRF1 depletion reduced association of eRF3 and eIF3 with 80S ribosomes isolated from heavy polysomes. The *Tet::SUP45* cells were grown in SD medium at 30°C in the presence or absence of 1 µg/ml doxycycline for six hours before harvesting, and treated as described in Figure 4A with the exception that only heavy polysomes were collected after the first round of centrifugation, and only the 80S couples were collected after the second round of centrifugation. Thus obtained samples were subsequently subjected to Western blot analysis; this experiment was conducted three times.

To provide more solid evidence implicating eIF3 and HCR1 in the process of termination *in vivo*, we tested whether or not both factors associate with polysomal 80S ribosomes by separating the formaldehyde cross-linked whole cell extracts (WCEs) on sucrose gradients by high velocity sedimentation into two major polysomal pools; the first containing light polysomes (disomes and trisomes) and the other heavy polysomes (from pentasomes up). These two pools were then treated with RNase I (Invitrogen) to chop polysomal mRNAs into segments containing either initiating 43S-48S PICs or elongating/terminating 80S ribosomes. The second round of sucrose gradient centrifugation (so called resedimentation; [25]) was employed to separate the 43-48S PICs from 80S couples in each polysomal pool into two fractions, which were then subjected to Western blotting. In both pools, the 80S fractions contained more than 50% of total eIF3 in comparison with the 40S fractions (Figure 4A), clearly demonstrating that only the lesser proportion of eIF3 occuring in polysomes is associated with initiation complexes. Strikingly, in case of HCR1, the 80S fractions contained even more, ∼90% of this protein from the overall pool. In contrast, the “polysomal” fraction of eIF5, which is known to tightly interact with eIF3 during translation initiation, was predominantly associated with 40S species. These results are thus consistent with a role of eIF3 and HCR1 in other translational phases than just initiation.

In the resedimented light and heavy polysomes different ratios of terminating versus initiating plus elongating ribosomes can be expected based on the following arguments. Under nutrient-rich conditions, the disome/trisome fraction will contain short mRNAs that cannot accommodate more than two/three ribosomes, poorly translated mRNAs, as well as recently transcribed mRNAs, which are not yet in a steady state phase with regards to their ribosome occupancy. Since mRNAs shorter than 60 codons make up only 2% of the yeast transcriptome [27], we expect that a majority of mRNAs in this pool are newly synthetized species with a standard/average mRNA length. Hence we posit that the light polysomal mRNAs contain a smaller proportion of terminating ribosomes than mRNAs isolated from heavy polysomes, since the likelihood of having a stop codon occupied by a terminating ribosome increases with the increasing number of elongating ribosomes per mRNA. In support of this rationale, we observed more than 3-fold increase in the amounts of eRF3 associated with 80S ribosomes isolated from heavy versus light polysomes. By the same token, if eIF3 was involved specifically in translation termination events, we would expect stronger association of eIF3 and HCR1 with 80S couples originating from heavy polysomes. We do indeed observe that the 80S/40S ratio of eIF3 and HCR1 association is 2- and 2.5-fold higher, respectively, for heavy polysomes than for the light ones, in contrast to eIF5 where it remains the same (Figure 4A). These results thus strongly suggest that eIF3 and HCR1 are present at 80S ribosomes during the terminating process.

To further support this conclusion, we employed the *Tet::SUP45* conditional depletion strain. We rationalized that if eIF3 specifically associates with terminating ribosomes, depletion of eRF1 should significantly reduce the presence of eIF3 subunits (as well as the presence of eRF3) in the 80S couples isolated from heavy polysomes. To test this, we formaldehyde cross-linked the *Tet::SUP45* cells grown in the presence or absence of 1 µg/ml doxycycline for six hours before harvesting, resolved the WCEs on sucrose gradients, collected only the fractions containing heavy polysomes, treated these fractions with RNase I and separated the resulting 43-48S PICs and 80S species by the second round of centrifugation (resedimentation). Thus isolated 80S couples from Dox^−^ versus Dox^+^ *Tet::SUP45* cells were loaded in six serial two-fold dilutions to the SDS-PAGE gel and the amounts of RPS0A (as a loading control) and associated eIFs and eRFs were analyzed by quantitative western blotting. Depletion of the key termination factor had to be rapid to avoid disassembly of stalled post-TCs in polysomes as well as secondary defects of dying cells. In our set-up we achieved ∼70% depletion of eRF1 and observed no changes in polysome profiles of Dox^−^ versus Dox^+^ *Tet::SUP45* cells (data not shown). As predicted, whereas the 80S-associated amounts of eIF1 remained unchanged (small), the amounts of eRF3 and two eIF3 subunits were significantly reduced (by ∼40%) in Dox^+^ versus Dox^−^ *Tet::SUP45* cells (Figure 4B). Note that while the overall levels of eRF1 were depleted by ∼70%, polysome-associated eRF1 was only depleted by ∼30%, which is consistent with the quantitatively similar reduction in polysome association observed for eRF3 and eIF3.

### Deletion of *hcr1* results in accumulation of eRF3 in heavy polysomes, and the *sup45^Y410S^* mutant prevents stable association of eRF3 and HCR1 with polyribosomes

In order to examine how the network of interactions between translation initiation and termination factors affects their functions, we investigated the distribution of selected translation factors in wt cells and cells mutated for either of the factors under study using formaldehyde cross-linking of living cells by sucrose density gradients analysis of WCEs [25].

**Figure 5:**
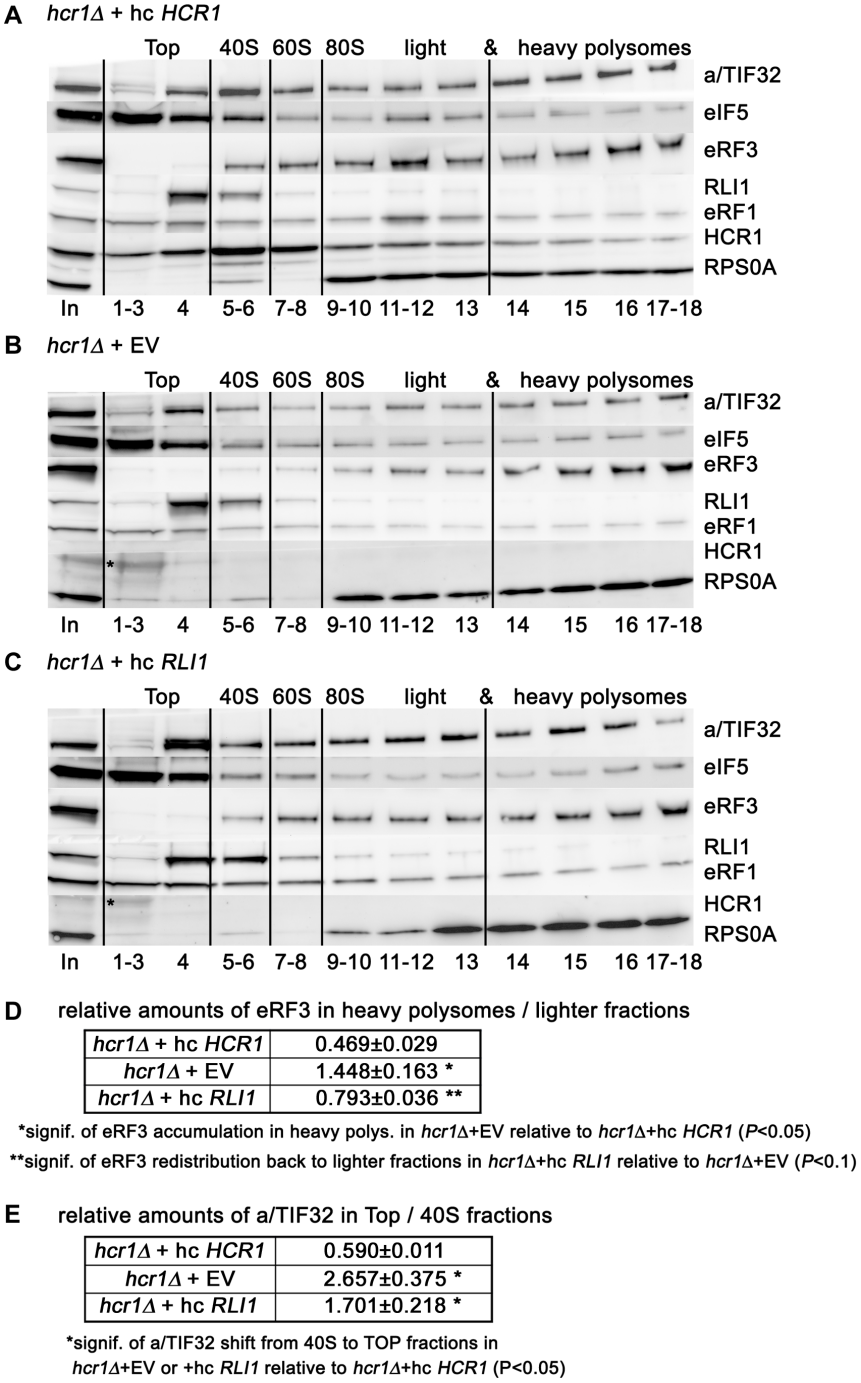
Deletion of *hcr1* results in accumulation of eRF3 in heavy polysomes. (**A–C**) The *hcr1Δ* strain (H3675) was transformed with either hc *HCR1* (**A**), empty vector (**B**), or hc *RLI1* (**C**), and the resulting transformants were grown in SD medium at 30°C to an OD_600_ of ∼1 and cross-linked with 0.5% HCHO prior to harvesting. WCEs were prepared, separated on a 5%–45% sucrose gradient by centrifugation at 39,000 rpm for 2.5 h and subjected to Western blot analysis. Several fractions corresponding to the Top, 40S, 60S, and 80S plus polysomal species were pooled, as indicated. Asterisk indicates a non-specific band. (**D**) Statistical significance of the eRF3 accumulation in heavy polysomes in the *hcr1* strain and its partial recovery by hc *RLI1*. Amounts of each individual factor in all fractions were quantified by fluorescence imaging. Thus obtained values for the fractions containing heavy polysomes (14–18) as well as all remaining fractions (1–13) were added up for each of these two groups. Values (mean±SE; n = 4) given in the table then represent relative amounts of factors in heavy polysomes divided by the compound value of the rest of the gradient. Changes in the redistribution of factors between the heavy polysomes and lighter fractions in all three strain were analyzed by the student's *t*-test and shown to be statistically significant only for eRF3 as shown in the table. (**E**) Statistical significance of the eIF3 shift from 40S-containing fractions to the top, which is independent of the effect of hc *RLI1* on eRF3. Essential the same as in panel D, except that the values for the Top fractions (1–4) as well as the 40S fractions (5–6) were added up for each of these two groups. Values (mean±SE; n = 4) given in the table then represent relative amounts of factors in the Top divided by the 40S group. Changes in the redistribution of factors between the 40S and Top fractions in *hcr1Δ*+EV or +hc *RLI1* strains vs. wt were analyzed by the student's *t*-test and shown to be statistically significant only for eIF3 as shown in the table.

Figures 5A and S7A show a typical distribution of the selected proteins across all gradient fractions obtained from wt WCEs and divided into several separable groups: “Top” (fractions 1–4), “40S” (5–6), “60S” (7–8), “80S+light polysomes” (9–13) and “heavy polysomes” (14–18). For technical reasons, several fractions from individual groups were pooled together to fit all samples on a single SDS-PAGE gel. Whereas eRF3 is clearly enriched in the polysome-containing fractions and practically lacking in the Top fractions, eRF1 is more or less evenly distributed across the entire gradient, and RLI1 predominantly sediments in the Top fractions and partially also in the 40S-containing fractions. Importantly, all strains that we worked with in this study are [psi^−^] and hence the observed sedimentation of eRF3 into heavier fractions cannot be attributed to SUP35 aggregation. In agreement with the aforementioned analysis, eIF3 (represented by a/TIF32) and HCR1 show a robust enrichment in polysomal fractions, similar to eRF3, whereas eIF5 occurs mainly in the Top and 40S fractions.

**Figure 6:**
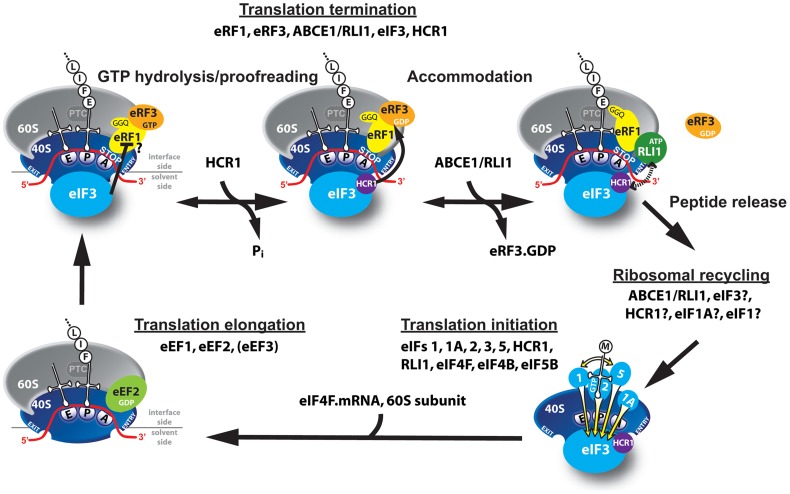
Model of eIF3 and HCR1 involvement in yeast translation termination. Upon stop codon entry into the ribosomal A-site the pre-TC forms, composed of the canonical release factors eRF1 and eRF3·GTP, and eIF3 and HCR1. eRFs and eIF3 may associate with the pre-TC as a pre-formed unit or alone. In the pre-TC, eIF3 interacts with the N domain of eRF1, *via* its two small g/TIF35 and i/TI34 subunits, and modulates, perhaps inhibits its stop codon recognition activity during the proofreading step. Upon stop codon recognition the GTP molecule on eRF3 is hydrolyzed. Subsequently, HCR1 promotes eRF3·GDP ejection to allow the ABCE1/RLI1·ATP recruitment to begin the accommodation phase of termination – the eRF1 GGQ motif is pushed to the peptidyl-transferase center (PTC) – during which HCR1 interacts with ABCE1/RLI1. Subsequently, both factors together with eIF3 participate in ribosomal recycling to enable and promote initiation of the next translational cycle (the elongation step is shown only for illustration purposes).

As shown in Figures 5B, E and S7A, deletion of *hcr1* significantly shifted the amounts of “initiating” eIF3 from the 40S fractions to the Top, as observed before [20], whereas it had no effect on polysomal distribution of eRF1 and RLI1. However, it led to a statistically significant accumulation of eRF3 in heavy polysomes with a commensurate reduction in lighter fractions (Figures 5B, D and S7A). We interpret the accumulation of eRF3 in heavy polysomes as an increased number of post-TCs bound by eRF3 in a less-productive manner; perhaps with a decreased dissociation rate. Most importantly, overexpression of RLI1, which suppresses both the read-through and Slg^−^ phenotypes of *hcr1Δ* (Figure 2), partially but significantly restored the eRF3 distribution in polysomes to wt (Figures 5C, D and S7A). The fact that the 40S-binding by the “initiating” eIF3 was not restored (Figures 5C, E and S7A) underscores a specificity of the RLI1 suppressor effect on the HCR1 role in termination versus initiation. One way to explain these observations is that HCR1 may promote the release of eRF3·GDP from the post-TCs upon stop codon recognition and GTP hydrolysis on eRF3, which serves as a prerequisite for the subsequent binding of RLI1 as well as the eRF1-stimulated hydrolysis of the bond between the P-site tRNA and the nascent polypeptide (see our model in Figure 6). Inability to complete this step may lead to a reduced stop codon recognition resulting in an increased read-through, which was observed. Hence the suppression effect of RLI1 on the molecular level could be explained by proposing that increased dosage of RLI1 forces dissociation of eRF3·GDP from the post-TCs by mass action and thus eliminates a need for HCR1.

**Figure 7:**
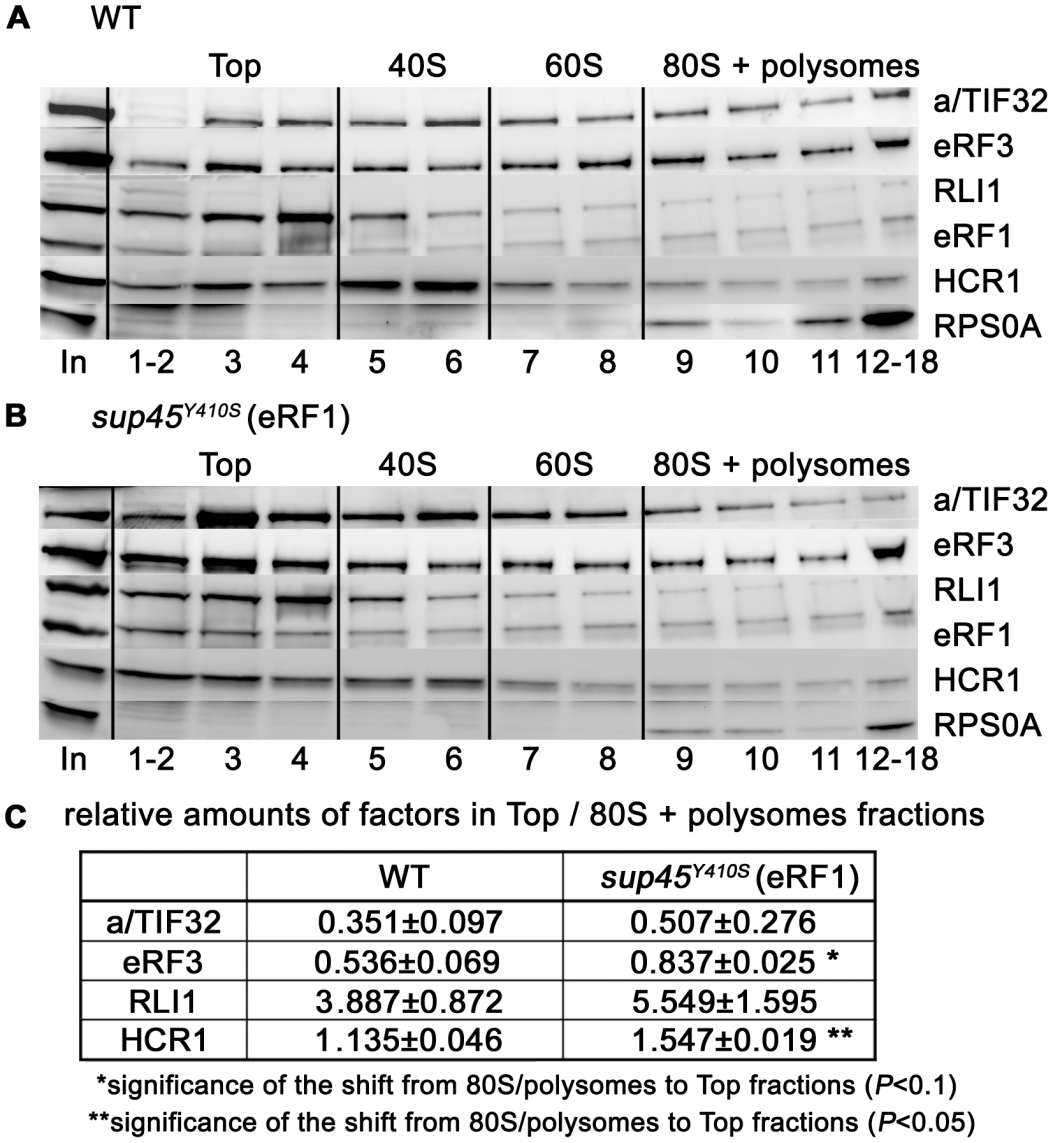
The *sup45^Y410S^* mutation prevents stable association of eRF3 and HCR1 with polyribosomes. (**A–B**) The *sup45^Y410S^* mutant and its corresponding wt strain were subjected to HCHO cross-linking (0.5%) and polysomal gradient analysis as described in Figure 5. (**C**) Statistical significance of the reduction of polysomes-associated amounts of eRF3 and HCR1 in *sup45^Y410S^*. Amounts of each individual factor in all fractions were quantified by fluorescence imaging. Thus obtained values for the Top fractions as well as fractions containing 80S couples and polysomes were added up for each of these two groups. Values (mean±SE; n = 4) given in the table then represent relative amounts of factors in the Top divided by the 80S+polysomes group. Changes in the redistribution of factors between the Top and 80S+polysomes fractions in the *sup45^Y410S^* mutant vs. wt were analyzed by the student's *t*-test and shown to be statistically significant for HCR1 (*P*<0.05) and SUP35 (*P*<0.1).

If our model is correct, one can predict that the polysomal levels of HCR1 could be reduced without any functional defect, if the interaction between eRF1 and eRF3 was impaired. Hence we next analyzed changes in polysomal distribution of factors of interest in the *sup45^Y410S^* mutant, which disrupts the eRF1–eRF3 interaction [28]. As expected, the *sup45^Y410S^* mutant significantly shifted the amounts of eRF3 from polysomal to the Top fractions when compared to wt (Figures 7A–C and S7B). Importantly, in accord with our proposed model, a similar, significant change was also observed for HCR1 but not for a/TIF32 and RLI1. We interpret these data by proposing that HCR1 readily dissociates along with eRF3 when eRF3 binding to the post-TCs is weakened by a mutation. This effect could be either direct or indirect/allosteric. The fact that we could not detect any direct binding between HCR1 and eRF3 using conventional *in vitro* protein-protein binding techniques may speak for the latter option; however, it is also possible that HCR1–eRF3 binding does occur but only in the context of the post-TCs.

### Genetic interactions between *hcr1Δ*, mutant eIF3 subunits and mutant release factors

To further support our model, we analyzed genetic interactions between the *hcr1* deletion strain and selected mutations in both release factors. The temperature sensitive eRF mutants we used are all known to cause termination defects including stop codon read-through strong enough to suppress the *ade1-14* nonsense allele [29]. They include a *sup35^N536T^* mutant located in a region near the C-terminus of eRF3 that disrupts termination by an unknown mechanism, a *sup45^M48I^* mutant that interferes with stop codon decoding [30], and the aforementioned *sup45^Y410S^* mutant that directly disrupts the eRF1–eRF3 interaction [28].

**Figure 8:**
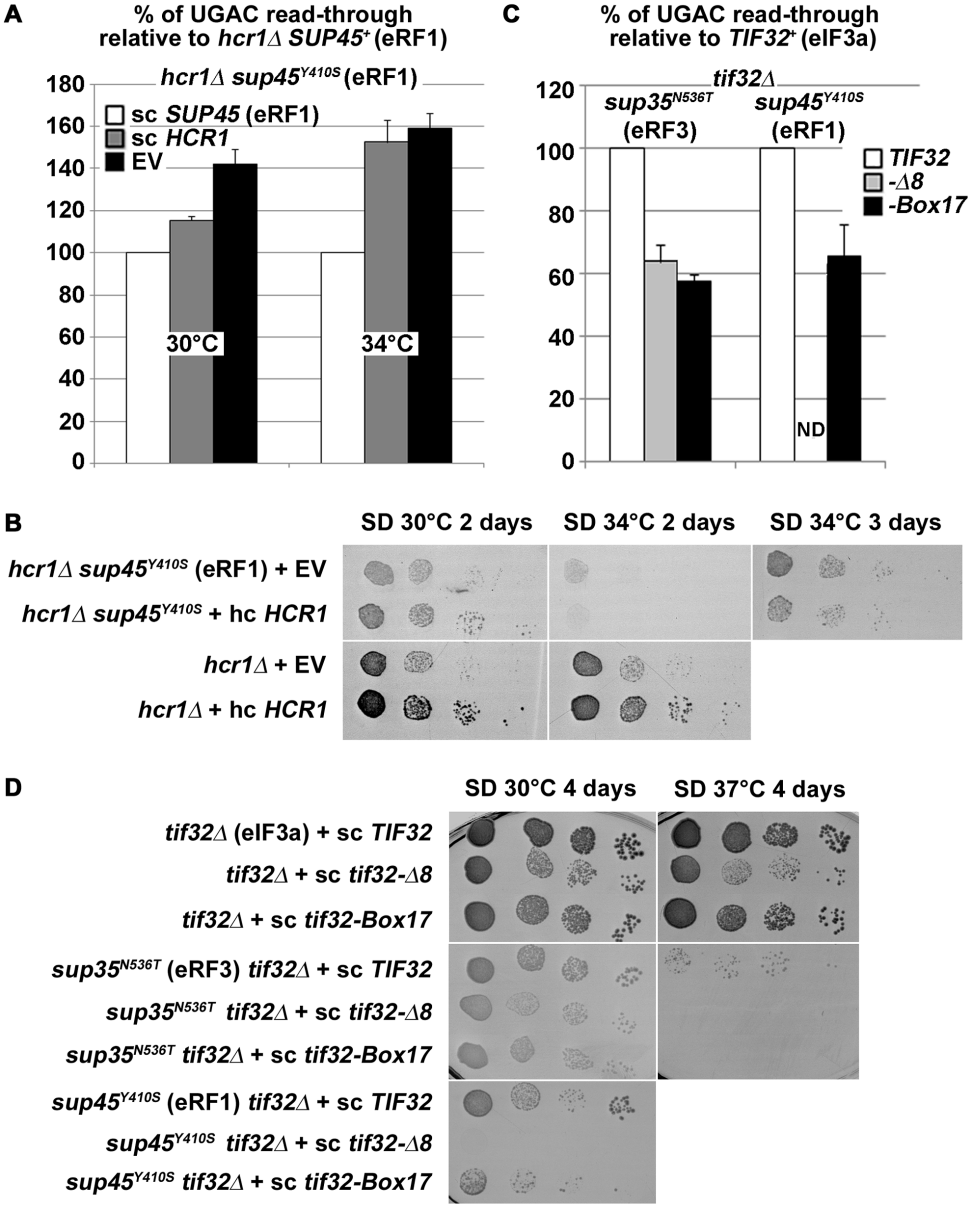
*hcr1Δ* and eIF3 mutants genetically interact with release factor mutants. (**A–B**) The *sup45^Y410S^* mutation eliminates the negative impact of *hcr1Δ* on (**A**) read-through and (**B**) growth rates. The *hcr1Δ* strain was crossed with the *sup45^Y410S^* mutant strain and the resulting double mutant was transformed with sc *SUP45*, hc *HCR1*, or empty vector (EV), respectively, and (**A**) processed for stop codon read-through as described in Figure 1 (*hcr1Δ* read-through values were set to 100%) or (**B**) subjected to a growth spot assay at indicated temperatures for 2 or 3 days. (**C–D**) Combining the selected *TIF32* mutants with *sup35^N536T^* and *sup45^Y410S^* (**C**) reduces their read-through defects and (**D**) produces synthetic growth phenotypes. The wt and mutant alleles of *TIF32* were introduced into *tif32Δ*, *sup35^N536T^ tif32Δ*, and *sup45^Y410S^ tif32Δ* strains, respectively, by plasmid shuffling. (**C**) The resulting double mutant strains were grown in SD and processed for the stop codon read-through as described in Figure 1 (the read-through values of both single eRF mutants were set to 100%), or (**D**) spotted in four serial 10-fold dilutions on SD medium and incubated at indicated temperatures for 4 days. ND; not determined due to severe growth deficiency.

It could be proposed that if the *sup45^Y410S^* Ts^−^ mutant reduced or even eliminated a need for HCR1 functioning in termination, an epistatic interaction should be observed when this mutant is combined with *hcr1Δ*. Consistently, at the permissive temperature the absence of HCR1 further increased read-through of this *sup45* mutant (Figure 8A, 30°C; compare open and grey bars with the black one) and also exacerbated its slow growth (Figure 8B). However, at the higher temperature, where the eRF1:eRF3 interaction is more severely disrupted by the *sup45^Y410^* mutation [28], as evidenced by its increased termination defect (Figure 8A; compare grey bars between 30 and 34°C), the absence of HCR1 had only a little additional effect on the *sup45^Y410S^* read-through (Figure 8A, 34°C; black vs. grey bars). Moreover, the *sup45^Y410S^* mutation also completely eliminated the negative impact of *hcr1Δ* on growth rates at this temperature (Figure 8B). The specificity of this epistatic interaction is further underscored by the fact that neither *sup45^M48I^* (eRF1) nor *sup35^N536T^* (eRF3) mutants showed any synthetic effects in the background of the *hcr1* deletion (Figure S7C).

Finally, to obtain further genetic evidence supporting our findings implicating eIF3 in regulation of the termination process, we combined two selected mutations in the a/TIF32 subunit of eIF3 (*Δ8* and *Box17*), both reducing the stop codon read-through in otherwise wt cells (Figure 1), with the *sup35^N536T^* and *sup45^Y410S^* mutants. When combined, the double mutants show a stop codon read-through frequency that is clearly reduced compared to either release factor mutant (Figure 8C), demonstrating that the *tif32* mutations partially rescue the read-through phenotype of the latter. In contrast, when we investigated slow growth (Slg^−^) and temperature sensitive (Ts^−^) phenotypes, we observed synthetic exacerbation of these phenotypes (Figure 8D). This demonstrates that i) the release factors and the core eIF3 complex have antagonistic functions in the same stage of the termination phase and losses in their functions can thus partially compensate for each other in terms of the stop codon read-through efficiency; and ii) that the degree of stop codon read-through *per se* is not the major source of the fitness defects in these strains. This latter notion is consistent with earlier quantitative trait analyses, which showed that the termination defects are unlinked from growth defects in many eRF1 mutants [31]. Hence, synthetic exacerbation of growth could be explained by proposing that besides the stop codon recognition step (which is assessed in the dual luciferase assay), also other aspects of termination are impaired in the eRF and eIF3 mutants, which, in combination with the initiation defects of eIF3 mutants, reduce the growth rate as a compound effect.

## Discussion

It is becoming increasingly apparent that factors involved in regulating various steps of gene expression may have multiple functions and that this multitasking may integrate transcription, mRNA export, translation and mRNA decay into a delicately regulated higher-order process. For example, translation initiation factor eIF3 links translation initiation to transcription [32], to mRNA export [3] and to the NMD pathway [33], [34]. Here we show for the first time that eIF3 and HCR1 critically connects initiation of translation with its termination.

In particular, deletion of *hcr1* increases stop codon read-through, independently of its association with the rest of eIF3, and results in accumulation of eRF3 in heavy polysomes. Increased dosage of ribosomal recycling factor RLI1 then substitutes for the HCR1 roles in termination (but not in initiation) and in enabling efficient cell growth, together implying that the HCR1 function in termination is more critical for optimal cell proliferation than its function in translation initiation. This is consistent with the fact that yeast HCR1 is only loosely associated with the core eIF3 complex [19] and that it was shown to interact with both sides of the 40S mRNA entry channel on its own [21]. Similarly, its mammalian ortholog also appears to be the most loosely associated subunit of all 13 eIF3 subunits that is, in addition, often missing from the purified 12-subunit complex [35]–[37]. Moreover, it was also shown to associate with the 40S ribosome completely independently of the rest of eIF3 and promote several translational steps practically on its own (see for example [5], [35]–[40]). Taken together, we suggest considering eIF3j/HCR1 as an independent initiation factor (eIF) that associates and closely co-operates with eIF3 but it is not its integral part. We therefore propose to use the following designations for this old-new eIF: HCR1 for the yeast protein and hHcr1 for its mammalian counterpart.

In contrast to *hcr1Δ*, various mutants of core eIF3 subunits, but not of other initiation factors, decrease stop codon read-through in living cells and show synthetic phenotypes with mutant release factors eRF1 and 3. eIF3 also directly interacts with eRF1 and occurs in complex with eRF1, eRF3 and RLI1 *in vivo*. Since eIF3 and HCR1 were, based on *in vitro* experiments, previously implicated in promoting also the very final step of translation – ribosomal recycling [5], we propose that eIF3 – and to some extend also HCR1 – is one of the very few factors that connects various processes of mRNA life and integrates them into the ultimate translational output. Taking into account that the translation pathway is highly conserved among low and high eukaryotes, it is highly likely that this connecting role of eIF3 is also conserved.

### eIF3 modulates the precision of the stop codon recognition by eRF1

Our observations that 1) eIF3 and eRF3 can be found enriched in 80S fractions isolated from RNase-treated heavy polysomes in an eRF1-dependent manner (Figure 4); 2) that a complex between eIF3, RLI1 and eRFs exists free of RNA and ribosomes in the cytoplasm (Figure 3A–D), and 3) that two small eIF3 subunits g/TIF35 (in particular its NTD) and i/TIF34 directly interact with the N and N-M domains of eRF1 (Figure 3E–F) together suggest that eIF3 does associate with terminating 80S couples and may come to the pre-TC in a pre-formed complex with eRFs. The alternative that they are ejected from post-TCs as a holocomplex upon completion of termination is highly unlikely considering that i) eRF3 must be ejected prior to RLI1 binding [12] and ii) that eIF3 is supposed to participate in the late steps of ribosomal recycling that should be devoid of eRF1 and RLI1 [5], [6]. The last scenario would be that eIF3 stays present on the elongating ribosome throughout the entire elongation cycle and promotes recruitment of eRF1·eRF3·GTP to the pre-TCs; there is, however, genetic evidence contradicting this possibility [9].

Our data show that eIF3 mutants specifically decrease stop codon read-through in otherwise wt cells (Figures 1 and S1) and that *tif32* mutations partially compensate for the increased read-through in eRF mutants (Figure 8C). This clearly suggests that wt eIF3 modulates the precision of stop codon recognition by eRF1 in order to fine tune the termination process (see our model in Figure 6). During stop codon decoding, eRF1 was proposed to sit in the ribosomal A-site with a part of its N-domain contacting small ribosomal protein RPS3 and helix (h) 18 of 18S rRNA [41]. Strikingly, g/TIF35 also interacts with RPS3, in addition to RPS20 [42], and as both g/TIF35 and i/TIF34 are tightly bound to the extreme C-terminus of b/PRT1 [43], i/TIF34 is expected to occur nearby g/TIF35. Moreover, the C-terminal domain of a/TIF32 interacts with h16-18 of 18S rRNA [44] and RPS3 as well [22]. Taylor and colleagues further proposed that one of the conformational changes induced by eRF1–eRF3–GMPPNP binding to pre-TCs involves a movement of h16 of 18S rRNA and the N-terminal domain (NTD) of RPS3 toward each other, which results in the establishment of a new head–body connection on the solvent side of the 40S subunit and a constriction of the mRNA entrance. Hence, it is conceivable that eRF1 and eIF3, by contacting the same 40S binding partners, modulate these conformational changes in the termination complex in a way that influences a proper placement of eRF1 in the spatially restricted A-site. This scenario could provide a rational explanation for the antagonistic effect of eIF3 on translation termination.

For interpretation of these data it must be kept in mind that the reporter constructs we use essentially measure stop codon read-through on a premature termination codon. At present, we do not know whether the antagonistic influence of eIF3 on stop-codon read-through is restricted to such sites, or whether it also affects termination on stop codons located nearer to the poly(A) tail. However, our observation that the *sup45^Y410S^* mutant, which affects stop codon selection by disrupting the eRF1–eRF3 interaction, reduced the polysome-associated amounts of eRF3 and HCR1 (Figure 7) indicates that a delay or imperfection in the decoding of natural stop codons disrupts this “initiation-termination” complex, most probably to enable resumption of elongation. Investigation of the precise molecular mechanism of the eIF3 action in termination is a pressing task for our future research.

### HCR1 promotes eRF3·GDP ejection from the post-TCs to allow RLI1 binding

In contrast to mutations in core eIF3 subunits, deletion of *hcr1* did not decrease but increased the stop codon read-through (Figure 1). The fact that mutations disrupting the HCR1 contact with eIF3 had no effect on read-through clearly suggests that the HCR1 role in termination is independent of its association with eIF3, as discussed above. Moreover, our findings that *hcr1Δ* results in accumulation of eRF3 in higher polysomal fractions (Figure 5) and that *sup45^Y410S^* (breaking the eRF1–eRF3 interaction) shifts HCR1 to the Top fractions (Figure 7) led to the model presented in Figure 6. We propose that following stop codon recognition and subsequent GTP hydrolysis on eRF3, HCR1 promotes eRF3·GDP ejection from the post-TCs to allow binding of its interacting partner RLI1 [2], which in turn stimulates polypeptide release – both eRF3 and RLI1 bind to the same site in the post-TC [13]. Inability to complete this step may lead to a reduced stop codon recognition resulting in an increased read-through. In support, eRF1 was shown to associate more firmly with post-TCs in the presence of eRF3 [6], which led the authors to propose that after GTP hydrolysis, eRF3 might not dissociate entirely from ribosomal complexes on its own and its release thus might require a stimulus by an additional factor; in our opinion by the HCR1 protein.

Based on the cryo-EM structures of DOM34:HBS1 (release factor-like proteins closely related in sequence and structure to eRF1:eRF3) on the yeast ribosome showing that the N-terminus of HBS1 extends away from the body of the protein and contacts the mRNA entry site, it was proposed that the N-terminus of eRF3 also occurs in the A-site area [13]. Since HCR1 was shown to occur in this area too [21], it could directly act upon this eRF3 domain to trigger the release of this factor in its GDP form from eRF1-bound post-TCs. In support, the N-terminal extension of *S. pombe* eRF3 was proposed to regulate eRF1 binding to eRF3 in a competitive manner [45]. Interestingly, both the N-terminus of eRF3 as well as the HCR1 protein as a whole are non-essential [45], [46], suggesting that they might act simply by shifting the equilibrium towards the loss of affinity between the eRF1 and eRF3·GDP binary complex. If true, the loss-of-function of both of them could be overcome by redundant mechanisms with slower reaction rates. In agreement, hc *RLI1* fully suppressed the read-through effect of *hcr1Δ* in a manner dependent on its intact 4Fe-4S and ABC domains (Figure 2B). We propose that in the *hcr1Δ* cells, RLI1 makes its way to its binding site in the post-TCs by forcing dissociation of eRF3·GDP through mass action and thus eliminates a need for HCR1. These results are consistent with the aforementioned observation that the eRF1 mutation *sup45^Y410S^*, disrupting the eRF1–eRF3 interaction, shifts the amounts of eRF3 and also that of HCR1 from polysomes to the top of the gradient (Figure 7).

The model proposed in Figure 6 also explains the behavior of genetic interactions observed for the *hcr1* deletion (Figure 8). Failure to eject eRF3·GDP can perceivably have two consequences. First, if peptidyl hydrolysis by eRF1 fails to be induced because RLI1 cannot bind to it, the eRF1·eRF3·GDP complex can dissociate from the ribosomal A-site, thus necessitating a renewed round of tRNA sampling with an ensuing risk of stop codon decoding by a near-cognate or suppressor tRNA. This is consistent with the increased stop codon read-through we observe experimentally in *hcr1* deletion strains. Second, if peptidyl hydrolysis does take place (*in vitro*, eRF1 clearly has some release factor activity also in the absence of RLI1 [11]), a stalled ribosome complex would be formed in which eRF1 was still bound to eRF3, and in which RLI1 was thus not free to initiate the recycling step. Such stalled complexes would impede ribosome flow on the affected mRNA, reduce corresponding gene expression levels and potentially necessitate degradation by one of the surveillance pathways. If this occurred frequently, it would give rise to fitness defects, as we observe for *hcr1* deletion strains. This is also consistent with the fact that deletion of *hcr1* produces unexpectedly mild polysomal run-off with respect to its growth defect [23]. However, in the presence of eRF1 mutations, which accelerate spontaneous dissociation of eRF3·GDP from eRF1, timely RLI1 binding to eRF1 in the post-TCs would be re-enabled even in the absence of HCR1. This would explain why the *sup45^Y410S^* mutation, but not *sup45^M48I^* and *sup35^N536T^* mutations, eliminated the negative impact of *hcr1Δ* on growth rates at the semipermissive temperature (Figure 8B).

To further support our model, we wished to employ a recently established *in vitro* reconstituted yeast translation system, which has been used previously to monitor both the peptide release and ribosome recycling steps of the translation cycle [12]. HCR1 did not have an appreciable effect on ribosome recycling in this assay (unpublished observations). This is probably not surprising given that ribosome recycling is slow relative to the preceding steps. Thus, accelerating eRF3.GDP dissociation is unlikely to affect the observed rate of recycling. In contrast, the model predicts that the observed rate of peptide release by eRF1, eRF3 and RLI1 may accelerate in the presence of HCR1. Unfortunately, however, the rate of peptide release by eRF1, eRF3 and RLI1 is very rapid, such that further increases in rate (such as those that may occur in the presence of HCR1) are unable to be measured in this system. Since the former two assays are the only *in vitro* assays available to us at the moment and neither of them can either directly or indirectly monitor the rate of eRF3·GDP dissociation from the post-TCs, further efforts will be necessary to fully characterize the role of HCR1 in termination/recycling reactions biochemically.

Upon completion of the termination-specific reactions, eIF3, HCR1 and RLI1 further participate in the ribosomal recycling steps, as proposed by [6], and it is conceivable that all these factors remain bound to the small 40S subunit to promote the next round of initiation (Figure 6). Alternatively, the pre-occupation of the 40S·mRNA complex by the “initiation factors” that would not be recycled could ensure reinitiation on the same mRNA molecule as proposed by the mRNA closed-loop model [47]. An *in vivo* experimental evidence implicating eIF3, HCR1 and other eIFs in the recycling steps is, however, still lacking.

### General conclusions

Taken together, we argue that strict mechanistic separation of translation into its individual, mutually independent phases should be reconsidered in the light of “multitasking” of eIF3, HCR1, RLI1 and most likely also eIF1 and eIF1A, for which evidence is presented here and elsewhere. Collectively, these findings suggest that changes in one phase of translation, evoked for example *via* cell signaling pathways, are promptly communicated to and coordinated with changes in the other phases to maintain cellular homeostasis of all ongoing processes. Without a doubt there is much to be learned about how all four phases of translation come together in one balanced system that rapidly and accurately responds to different needs of the cell exposed to constantly changing environmental conditions.

## Materials and Methods

### Yeast strains and plasmids

The lists and descriptions of plasmids and yeast strains used throughout this study can be found in the Supplemental Information (Tables S2, S3, S4 and Text S1).

### Read-through assays

Stop codon read-through assays were performed using a bicistronic reporter construct consisting of a *Renilla* luciferase gene followed by an in-frame firefly luciferase gene. Separating the two genes is either a tetranucleotide termination signal (e.g., UGA C) [plasmids pTH477 (*URA3*) or YEp-R/T-UGAC-L (*LEU2*)] or, for control purposes, a similar sequence containing a sense codon (e.g., CAA C) [plasmids pTH460 (*URA3*) or YEp-R/T-CAAC-L (*LEU2*)]. It is noteworthy that this system avoids possible artifacts associated with changes in the efficiency of translation initiation associated with the function of the NMD machinery [48], because both the *Renilla* and firefly enzymes initiate translation from the same AUG codon. For further details, see [14]. Microtitre-plate based dual luciferase assays and analyses of the resulting data were as described [31]. Samples were processed in quintuplicate, and each experiment was repeated at three times.

### Co-immunoprecipitation and affinity tag pull downs

Yeast whole cell extracts (WCEs) were prepared as described previously [49] except that buffer A (30 mM HEPES (pH 8.8), 20 mM KAc, 3 mM magnesium acetate,1 mM dithiothreitol, 1% Nonidet P-40 supplemented with Complete Protease Inhibitor Mix tablets (ROCHE), and protease inhibitors 1 µg/ml aprotinin, 1 µg/ml leupeptin, 1 µg/ml pepstatin and 100 µM phenylmethylsulfonyl fluoride (PMSF)) was used for lysis of the cells, and cell lysates were centrifuged at 3,000 r.p.m. for 10 min at 4°C. The co-immunoprecipitation analysis was performed as described elsewhere [50], using 500 µg of the total protein and 1 µl of mouse anti Myc-Tag IgG (CELL SIGNALING TECHNOLOGY).

Yeast cells expressing the TAP-tagged genes of interest were grown in YPD medium at 30°C to an OD_600_ of ∼1 and treated with 1% HCHO prior to harvesting for 60 mins. The WCEs was prepared as described above using buffer B (50 mM Tris-HCl (pH 7.6), 150 mM NaCl, 0.05% Tween 20) with all protease inhibitors in the presence or absence of 0.1 mg/ml RNase A. Samples containing 1 mg of total protein in a final volume of 600 µl were incubated for 2 h at 4°C with 50 µl of 1∶1 slurry of IgG Sepharose 6 Fast Flow beads in buffer B. Samples were centrifuged briefly and the supernatants were removed. The collected beads were then washed five times with 1 ml of ice cold buffer B, and incubated either with TEV protease (INVITROGEN) for 30 min at 30°C followed by boiling in the SDS-loading buffer for 5 min at 95°C, or directly boiled the SDS-loading buffer. Corresponding aliquots of input, eluate and wash (supernatant) were analyzed by SDS-PAGE followed by immunoblotting.

### Polysomal gradient analysis

The 0.5% formaldehyde (HCHO) cross-linking followed by WCE preparation and fractionation of extracts for analysis of translational complexes were carried out as described previously [25] with the following exceptions. Cycloheximide was added at a concentration of 0.05 mg/ml 5 minutes before the HCHO treatment, after which the cells were broken by FastPrep Instrument (MP Biomedicals) at the intensity level of 5 in two 20 second cycles. The resulting WCEs were separated on 5–45% sucrose gradients.

### Other yeast biochemical methods

GST pull-down experiments with GST fusions and *in vitro*-synthesized [^35^S]-labeled polypeptides (see Table S3 for vector descriptions) were conducted as follows. Individual GST-fusion proteins were expressed in *E. coli*, immobilized on glutathione-Sepharose beads and incubated with 10 µl of ^35^S-labeled potential binding partners at 4°C for 2 h. The beads were washed 3 times with 1 ml of phosphate-buffered saline, and bound proteins were separated by SDS-PAGE. Gels were first stained with Gelcode Blue Stain Reagent (Pierce) and then subjected to autoradiography. β-galactosidase assays were conducted as described previously [51].

### Preparation of antibodies against RLI1 and SUP45

The GST-RLI1 and GST-SUP45 fusion proteins encoded by pGEX-RLI1, pGEX-SUP45, respectively, were expressed in *E. coli* and purified from the WCE by incubation with Glutathione-Sepharose 4B beads (Pharmacia). The isolated proteins were resolved by SDS-PAGE (4–20% gels), excised from the gel, and washed with 1× PBS. Rabbits were injected with the purified protein and sera containing polyclonal antibodies against RLI1, SUP45, respectively, were obtained commercially by Apronex (Prague, the Czech Republic).

## Supporting Information

Figure S1 DaMP alleles of various 43S PIC-associated initiation factors display distinct effects on efficiency of stop codon read-through. Yeast strains containing kanMX4 cassettes integrated into their 3′-UTRs (so-called DaMP alleles) were recovered from the genome-wide collection for these alleles [15]. We were able to recover alleles for all 43S PIC-associated eIFs with the exception of eIF2β, for which no allele was present in the collection. In order to aid interpretation of results, and to assess the efficiency of depletion of the gene in question, we initially measured growth rates of the respective strains (top panel). Since all of the factors studied here are essential, we expected a reduction in growth rate upon significant depletion of any of these factors. We then proceeded to measure stop codon read-through in these strains, using dual luciferase reporters as described in the main text. Of the eIF3 subunits tested, only g/TIF35 is sufficiently depleted to cause a significant growth defect, and this strain shows a significant reduction in stop codon read-through. Moreover, the c/NIP1 DaMP allele also shows a significant reduction in stop codon read-through, even though this protein is not sufficiently depleted to produce a significant growth defect. Together, these results confirm those presented for other eIF3 alleles in the main text. In contrast to the eIF3 subunits, other 43S PIC-associated translation initiation factors do not reduce stop codon read-through upon depletion. Conversely, both eIF2 subunits tested and eIF1A increased read-through when depleted. This demonstrates that the role of eIF3 in translation termination is specific to this factor.
(TIF)

Figure S2 Increased gene dosage of eRFs 1 and 3 reduces stop-codon read-through. Wild type strain H416 was transformed with designated plasmids overexpressing eRFs and the resulting transformants were grown in SD and processed for the stop codon read-through measurements as described in Figure 1.
(TIF)

Figure S3 Increased gene dosage of eEF3 does not suppress the slow growth and read-through defects of *hcr1Δ*. (**A**) The *hcr1Δ* strain (H3675) was transformed with either empty vector, high copy (hc) *HCR1* or hc *YEF3* (eEF3), the resulting transformants were spotted in four serial 10-fold dilutions on SD medium and incubated at 30°C for 2 days. Unlike RLI1, eEF3 (which is also an ABC cassette-containing protein) does not suppress the growth defect of an *hcr1* deletion strain. (**B**) The strains from panel A were grown in SD and processed for the stop codon read-through measurements as described in Figure 1.
(TIF)

Figure S4 Increased gene dosage of HCR1 does not suppress the slow growth and read-through defects of the *Tet::RLI1* strain; intact ATP-binding cassettes and the Fe-S cluster of RLI1 are indispensable for its role in ensuring stop codon selection accuracy. (**A**) The *Tet::RLI1* (Tet-RLI1) and the corresponding wt strain (W303) were transformed with either empty vector or hc *HCR1*, and the resulting transformants were spotted in four serial 10-fold dilutions on SD medium supplemented with 0.25 µg/ml of doxycycline and incubated at 30°C for 2 days. (**B**) The *Tet::RLI1* (Tet-RLI1) strain was transformed with hc vectors carrying wt or mutant *RLI1* alleles, or empty vector or hc *HCR1*. The resulting transformants were grown in SD supplemented with 1 µg/ml of doxycycline (DOX) and processed for the stop codon read-through measurements as described in Figure 1. Obtained values were normalized to the value obtained with the *Tet::RLI1* strain transformed with wt *RLI1*, which was set to 100%.
(TIF)

Figure S5 Increased gene dosage of ABCE1/RLI1 does not suppress the leaky scanning defect of *hcr1Δ*. The *HCR1^+^* (H2879) and *hcr1Δ* (H3675) strains were first transformed with either empty vector or hc *RLI1* and subsequently with the *GCN4-lacZ* reporter plasmid plig102-3. The resulting double transformants were grown in SD medium at 30°C to an OD_600_ of ∼1. The β-galactosidase activities were measured in the WCEs and expressed in units of nmol of o-nitrophenyl-b-D-galactopyranoside hydrolyzed per min per mg of protein. The plots show mean values and standard deviations obtained from at least 3 independent measurements with three independent transformants. The fold-difference between the *hcr1Δ* versus *HCR1^+^* strains with or without hc *RLI1* is indicated.
(TIF)

Figure S6 Complexes containing eIF3, HCR1, ABCE1/RLI1 and both eRFs, free of ribosomes and RNA, occur *in vivo* – the RNase A treatment. (**A**) RNAse A-treated WCEs were prepared from HCHO-treated (1%) cells bearing wt (H2879) or TAP-tagged (H553) chromosomal alleles of *HCR1* and incubated with IgG Sepharose 6 Fast Flow beads. The immune complexes were eluted by boiling in SDS buffer and subjected to Western analysis. In, 1.5% of input; E, 50% of the elution fraction; W, 1.5% of the supernatant fraction. eRF1 is indicated by an asterisk below the immunoglobulins. (**B**) RNAse A-treated WCEs from HCHO-treated cells (1%) cells bearing wt (H2879) or TAP-tagged (H555) chromosomal alleles of *TIF32* were processed as in panel A except that the immune complexes were eluted by the TEV protease cleavage. In, 1.5% of input; E, 100% of the elution fraction; W, 1.5% of the supernatant fraction. (**C**) RNAse A-treated WCEs from HCHO-treated cells (1%) cells bearing wt (74D-694) or TAP-tagged (H517) chromosomal alleles of *SUP35* were processed as in panel B.
(TIF)

Figure S7 (**A**) Deletion of *hcr1* results in accumulation of the polysome-associated eRF3 – factor distributions across gradient fractions as shown in Figure 5. Amounts of each individual factor in the pooled fractions from three independent experiments were quantified by fluorescence imaging, combined, and the percentage representation of the signal corresponding to the Top (1–3 and 4), 40S (5–6), 60S (7–8), and 80S plus polysomal fractions (9 through 18) was calculated and plotted. (**B**) The *sup45^Y410S^* mutation prevents stable association of eRF3 and HCR1 with polyribosomes – factor distributions across gradient fractions as shown in Figure 7. Amounts of each individual factor in the pooled fractions from three independent experiments were quantified by fluorescence imaging, combined, and the percentage representation of the signal corresponding to the Top (1–3), 40S (4–5), 60S (6–7), and 80S plus polysomal fractions (8–11) was calculated and plotted. (**C**) The *sup45^Y410S^* but not the other mutations in eRFs 1 and 3 eliminate the negative impact of *hcr1Δ* on growth rates. The *hcr1Δ* strain was crossed with the indicated *sup45* and *sup35* mutant strains and the resulting double mutants (PBH104, PBH103 and PBH105) were transformed with either empty vector (EV) or hc vector containing *HCR1* and together with the corresponding *hcr1Δ SUP35 SUP45* “wt” strain (YLVH13) spotted in four serial 10-fold dilutions on SD medium and incubated at indicated temperatures for 2 or 3 days.
(TIF)

Table S1 Mutant alleles used in this study and their associated phenotypes.
(DOCX)

Table S2 Yeast strains used in this study.
(DOCX)

Table S3 Plasmids used in this study.
(DOCX)

Table S4 Primers used in this study.
(DOCX)

Text S1 Supporting Materials and Methods.
(DOCX)
