# Supplementary figures and images for: Translation Initiation Factors eIF3 and HCR1 Control Translation Termination and Stop Codon Read-Through in Yeast Cells
Source: PLoS Genet. 2013 Nov 21;9(11):e1003962. doi: 10.1371/journal.pgen.1003962 (PMC3836723; doi:10.1371/journal.pgen.1003962)

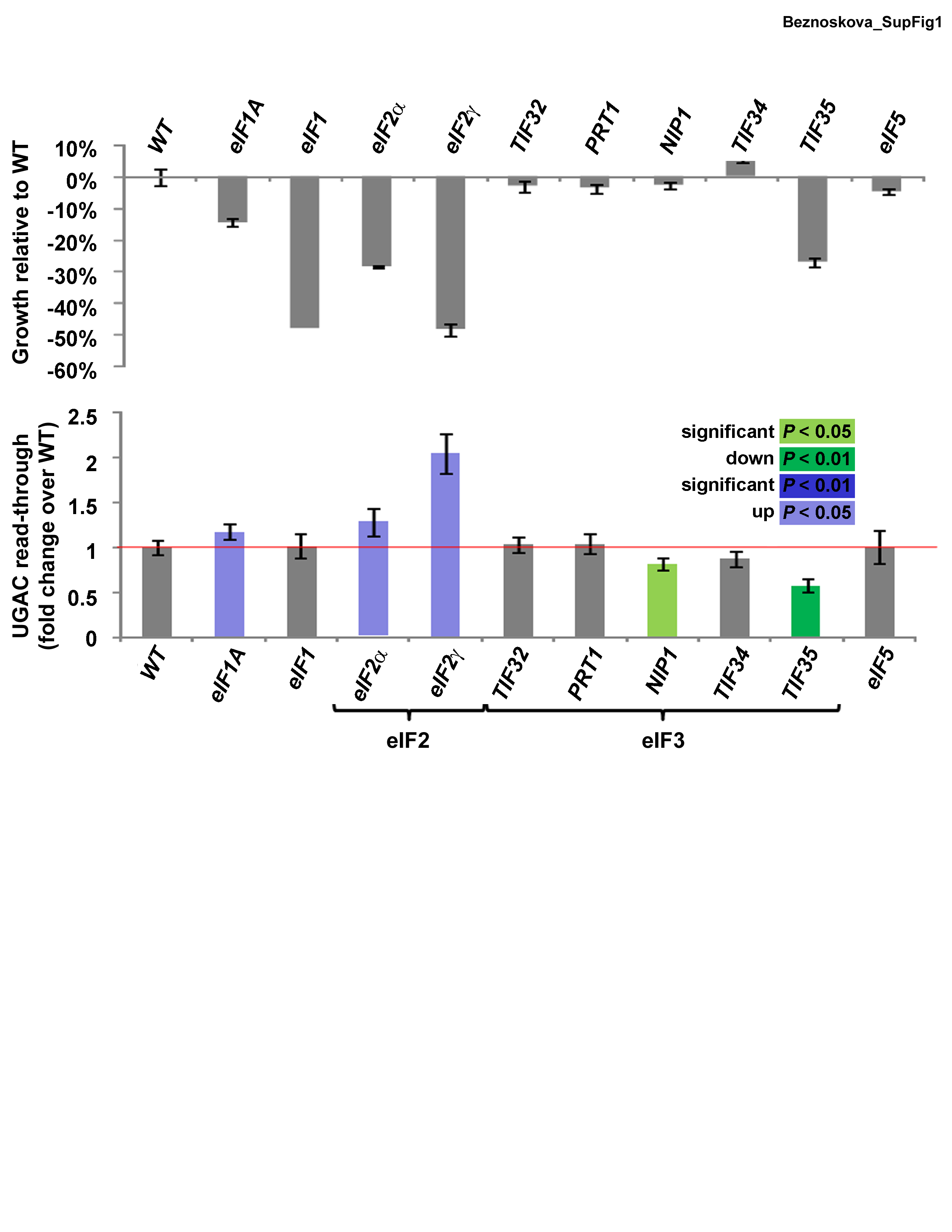

Supplement: Figure S1 — DaMP alleles of various 43S PIC-associated initiation factors display distinct effects on efficiency of stop codon read-through. Yeast strains containing kanMX4 cassettes integrated into their 3′-UTRs (so-called DaMP alleles) were recovered from the genome-wide collection for these alleles [15]. We were able to recover alleles for all 43S PIC-associated eIFs with the exception of eIF2β, for which no allele was present in the collection. In order to aid interpretation of results, and to assess the efficiency of depletion of the gene in question, we initially measured growth rates of the respective strains (top panel). Since all of the factors studied here are essential, we expected a reduction in growth rate upon significant depletion of any of these factors. We then proceeded to measure stop codon read-through in these strains, using dual luciferase reporters as described in the main text. Of the eIF3 subunits tested, only g/TIF35 is sufficiently depleted to cause a significant growth defect, and this strain shows a significant reduction in stop codon read-through. Moreover, the c/NIP1 DaMP allele also shows a significant reduction in stop codon read-through, even though this protein is not sufficiently depleted to produce a significant growth defect. Together, these results confirm those presented for other eIF3 alleles in the main text. In contrast to the eIF3 subunits, other 43S PIC-associated translation initiation factors do not reduce stop codon read-through upon depletion. Conversely, both eIF2 subunits tested and eIF1A increased read-through when depleted. This demonstrates that the role of eIF3 in translation termination is specific to this factor. (TIF) [file pgen.1003962.s001.tif]

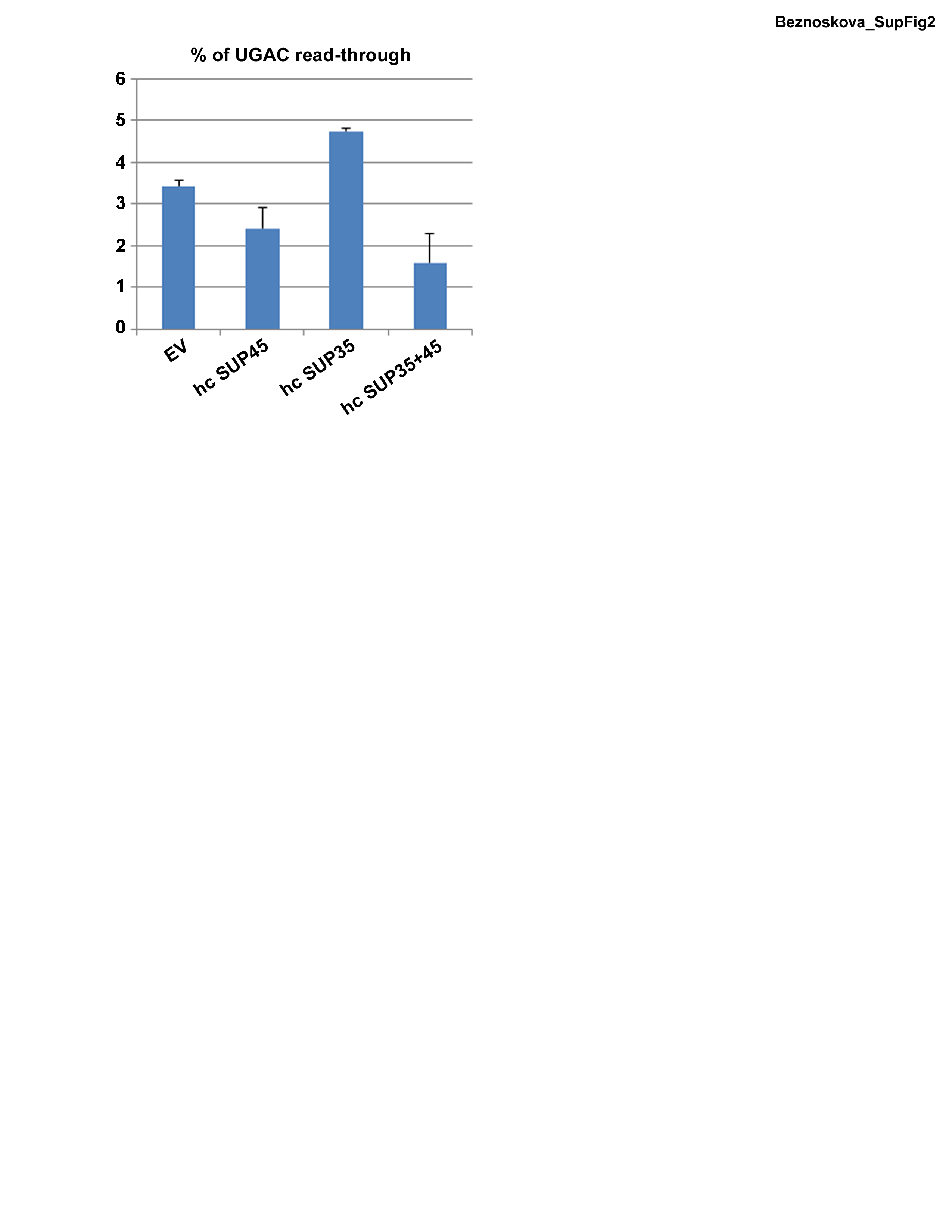

Supplement: Figure S2 — Increased gene dosage of eRFs 1 and 3 reduces stop-codon read-through. Wild type strain H416 was transformed with designated plasmids overexpressing eRFs and the resulting transformants were grown in SD and processed for the stop codon read-through measurements as described in Figure 1. (TIF) [file pgen.1003962.s002.tif]

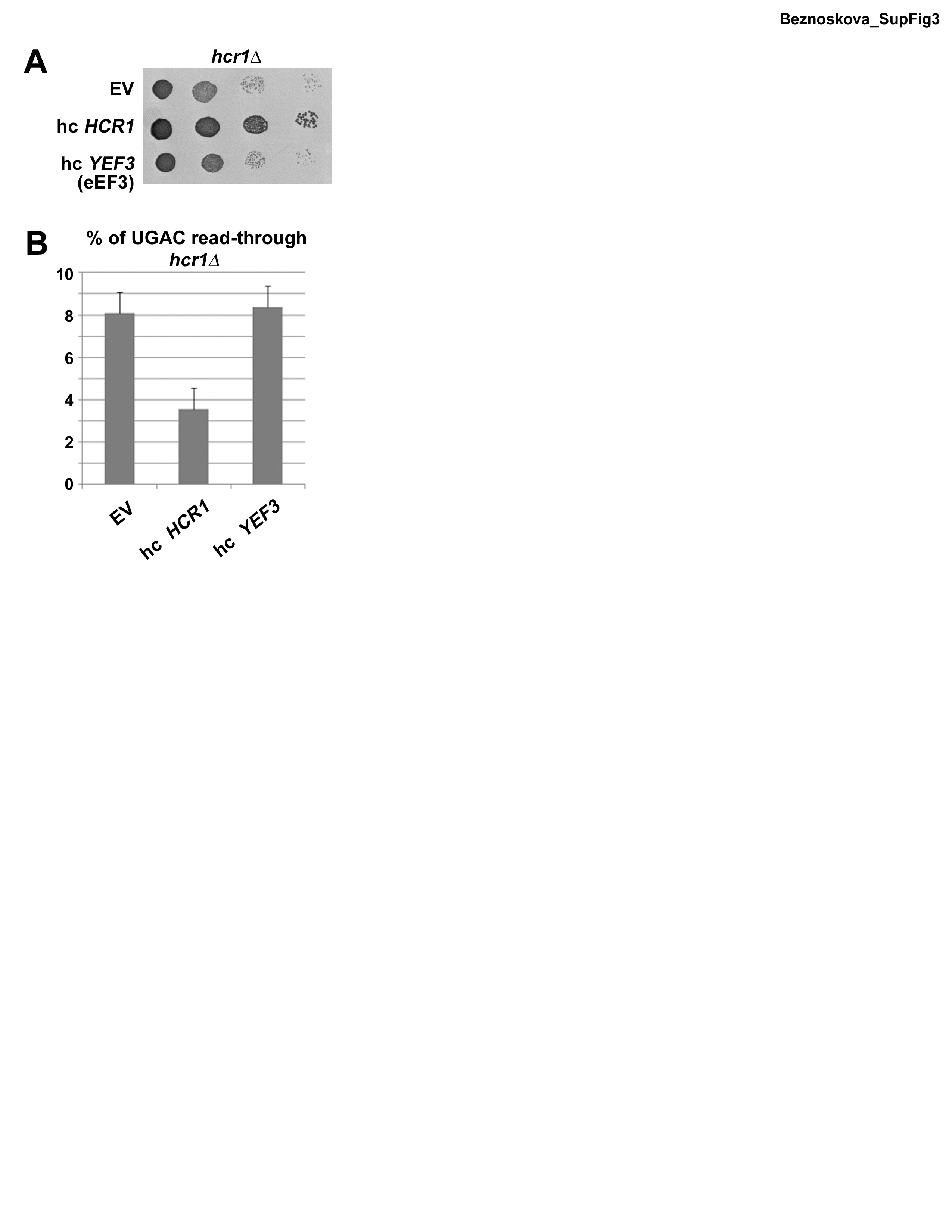

Supplement: Figure S3 — Increased gene dosage of eEF3 does not suppress the slow growth and read-through defects of hcr1Δ. (A) The hcr1Δ strain (H3675) was transformed with either empty vector, high copy (hc) HCR1 or hc YEF3 (eEF3), the resulting transformants were spotted in four serial 10-fold dilutions on SD medium and incubated at 30°C for 2 days. Unlike RLI1, eEF3 (which is also an ABC cassette-containing protein) does not suppress the growth defect of an hcr1 deletion strain. (B) The strains from panel A were grown in SD and processed for the stop codon read-through measurements as described in Figure 1. (TIF) [file pgen.1003962.s003.tif]

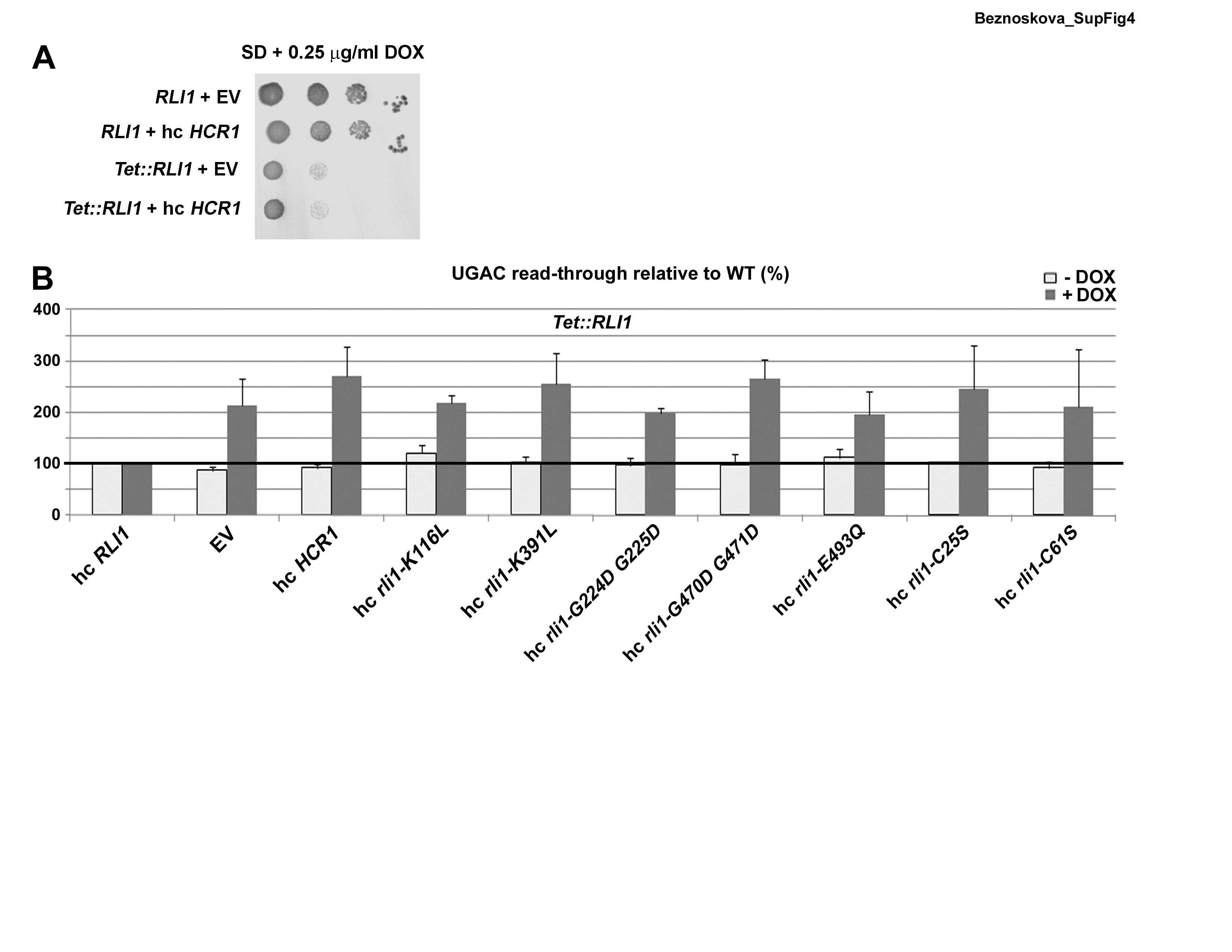

Supplement: Figure S4 — Increased gene dosage of HCR1 does not suppress the slow growth and read-through defects of the Tet::RLI1 strain; intact ATP-binding cassettes and the Fe-S cluster of RLI1 are indispensable for its role in ensuring stop codon selection accuracy. (A) The Tet::RLI1 (Tet-RLI1) and the corresponding wt strain (W303) were transformed with either empty vector or hc HCR1, and the resulting transformants were spotted in four serial 10-fold dilutions on SD medium supplemented with 0.25 µg/ml of doxycycline and incubated at 30°C for 2 days. (B) The Tet::RLI1 (Tet-RLI1) strain was transformed with hc vectors carrying wt or mutant RLI1 alleles, or empty vector or hc HCR1. The resulting transformants were grown in SD supplemented with 1 µg/ml of doxycycline (DOX) and processed for the stop codon read-through measurements as described in Figure 1. Obtained values were normalized to the value obtained with the Tet::RLI1 strain transformed with wt RLI1, which was set to 100%. (TIF) [file pgen.1003962.s004.tif]

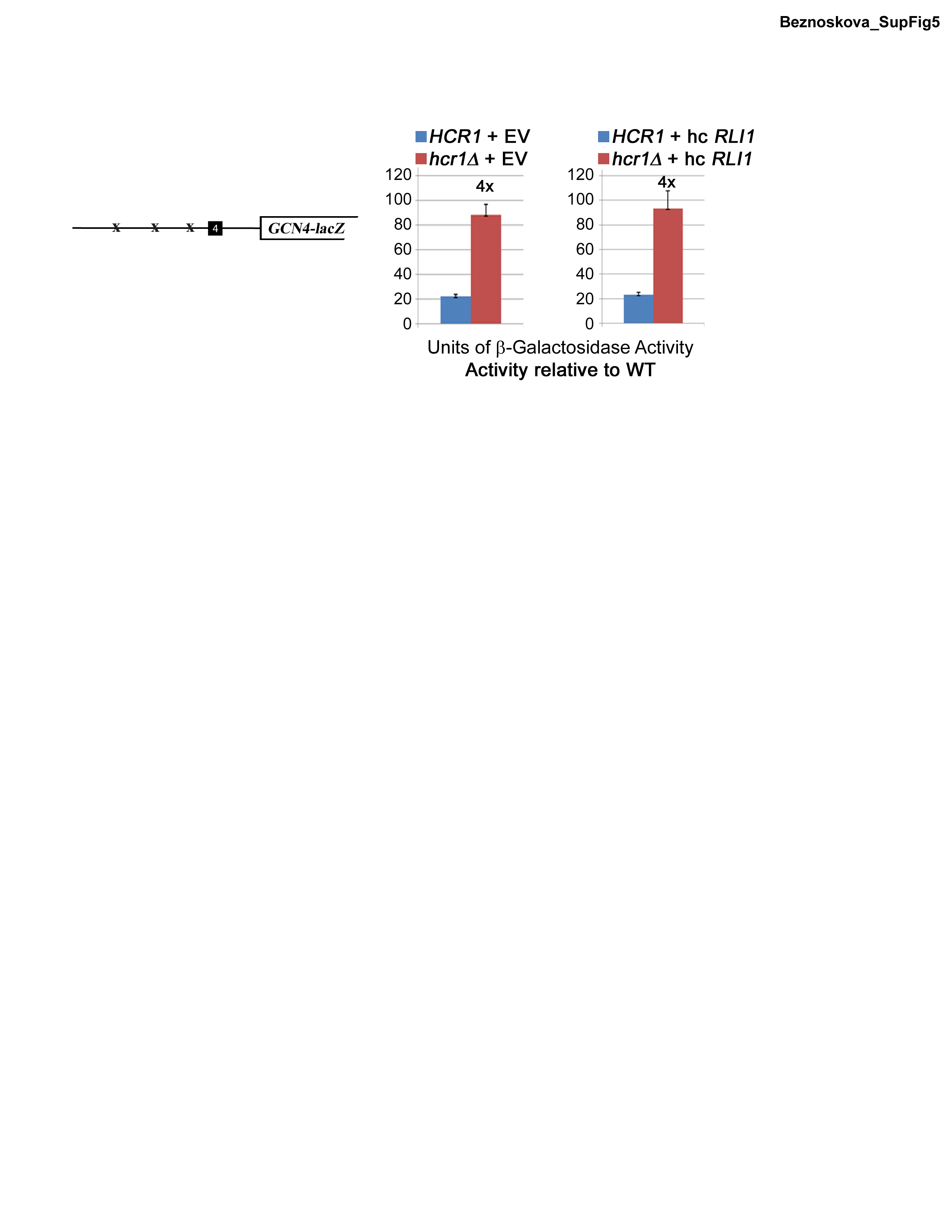

Supplement: Figure S5 — Increased gene dosage of ABCE1/RLI1 does not suppress the leaky scanning defect of hcr1Δ. The HCR1+ (H2879) and hcr1Δ (H3675) strains were first transformed with either empty vector or hc RLI1 and subsequently with the GCN4-lacZ reporter plasmid plig102-3. The resulting double transformants were grown in SD medium at 30°C to an OD600 of ∼1. The β-galactosidase activities were measured in the WCEs and expressed in units of nmol of o-nitrophenyl-b-D-galactopyranoside hydrolyzed per min per mg of protein. The plots show mean values and standard deviations obtained from at least 3 independent measurements with three independent transformants. The fold-difference between the hcr1Δ versus HCR1+ strains with or without hc RLI1 is indicated. (TIF) [file pgen.1003962.s005.tif]

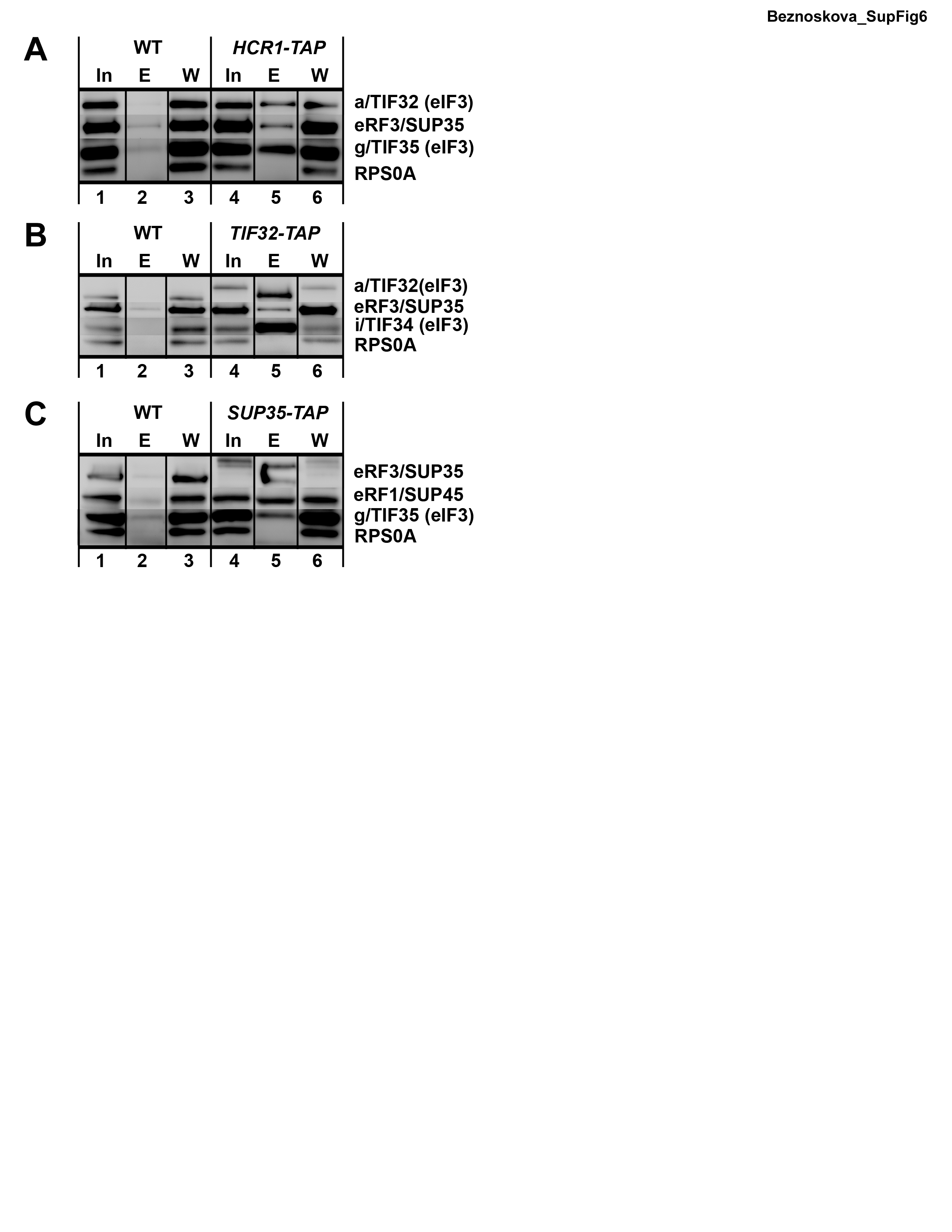

Supplement: Figure S6 — Complexes containing eIF3, HCR1, ABCE1/RLI1 and both eRFs, free of ribosomes and RNA, occur in vivo – the RNase A treatment. (A) RNAse A-treated WCEs were prepared from HCHO-treated (1%) cells bearing wt (H2879) or TAP-tagged (H553) chromosomal alleles of HCR1 and incubated with IgG Sepharose 6 Fast Flow beads. The immune complexes were eluted by boiling in SDS buffer and subjected to Western analysis. In, 1.5% of input; E, 50% of the elution fraction; W, 1.5% of the supernatant fraction. eRF1 is indicated by an asterisk below the immunoglobulins. (B) RNAse A-treated WCEs from HCHO-treated cells (1%) cells bearing wt (H2879) or TAP-tagged (H555) chromosomal alleles of TIF32 were processed as in panel A except that the immune complexes were eluted by the TEV protease cleavage. In, 1.5% of input; E, 100% of the elution fraction; W, 1.5% of the supernatant fraction. (C) RNAse A-treated WCEs from HCHO-treated cells (1%) cells bearing wt (74D-694) or TAP-tagged (H517) chromosomal alleles of SUP35 were processed as in panel B. (TIF) [file pgen.1003962.s006.tif]

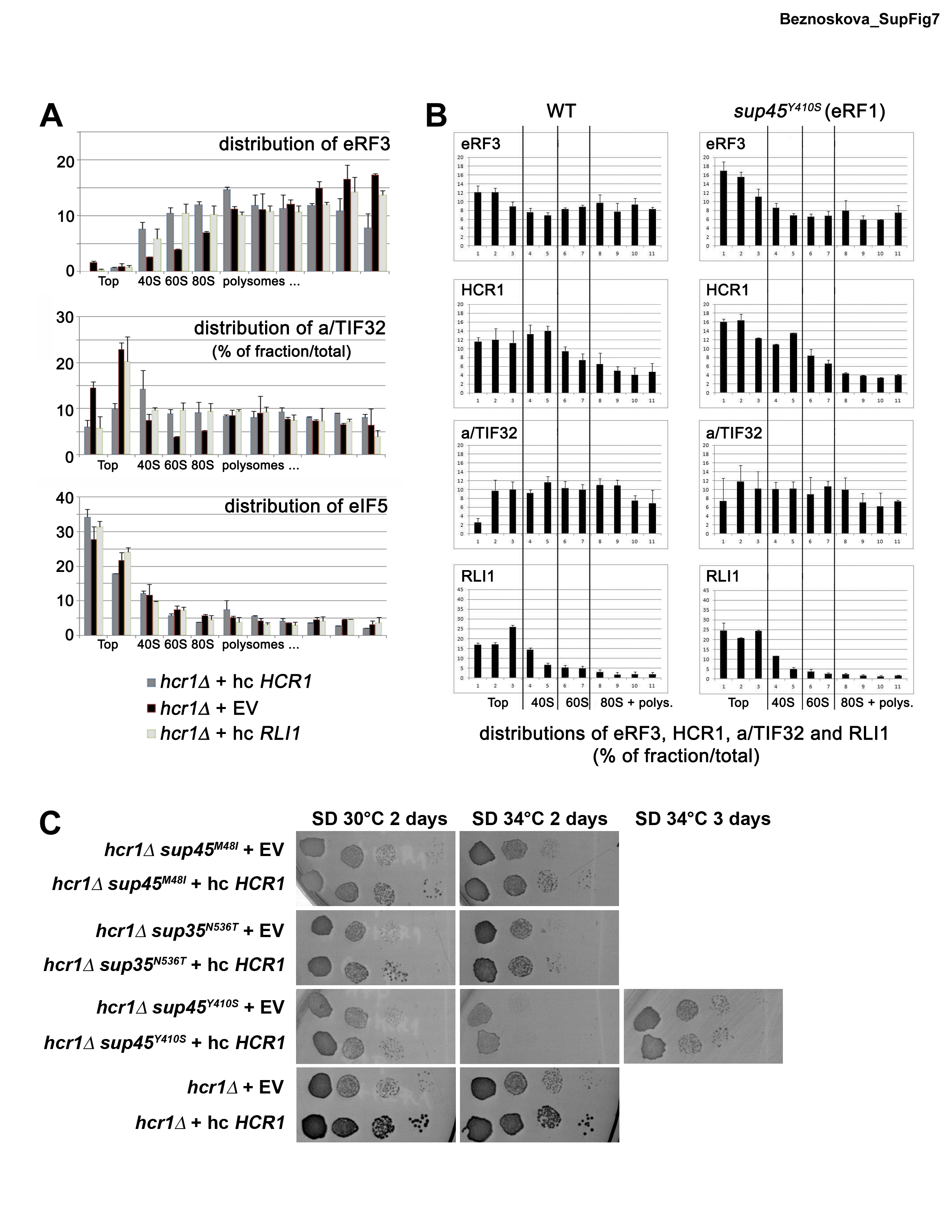

Supplement: Figure S7 — (A) Deletion of hcr1 results in accumulation of the polysome-associated eRF3 – factor distributions across gradient fractions as shown in Figure 5. Amounts of each individual factor in the pooled fractions from three independent experiments were quantified by fluorescence imaging, combined, and the percentage representation of the signal corresponding to the Top (1–3 and 4), 40S (5–6), 60S (7–8), and 80S plus polysomal fractions (9 through 18) was calculated and plotted. (B) The sup45Y410S mutation prevents stable association of eRF3 and HCR1 with polyribosomes – factor distributions across gradient fractions as shown in Figure 7. Amounts of each individual factor in the pooled fractions from three independent experiments were quantified by fluorescence imaging, combined, and the percentage representation of the signal corresponding to the Top (1–3), 40S (4–5), 60S (6–7), and 80S plus polysomal fractions (8–11) was calculated and plotted. (C) The sup45Y410S but not the other mutations in eRFs 1 and 3 eliminate the negative impact of hcr1Δ on growth rates. The hcr1Δ strain was crossed with the indicated sup45 and sup35 mutant strains and the resulting double mutants (PBH104, PBH103 and PBH105) were transformed with either empty vector (EV) or hc vector containing HCR1 and together with the corresponding hcr1Δ SUP35 SUP45 “wt” strain (YLVH13) spotted in four serial 10-fold dilutions on SD medium and incubated at indicated temperatures for 2 or 3 days. (TIF) [file pgen.1003962.s007.tif]
