# Supplementary material for: Translation Initiation Factors eIF3 and HCR1 Control Translation Termination and Stop Codon Read-Through in Yeast Cells
Source: PLoS Genet. 2013 Nov 21;9(11):e1003962. doi: 10.1371/journal.pgen.1003962 (PMC3836723; doi:10.1371/journal.pgen.1003962)
Supplement: Table S1 — Mutant alleles used in this study and their associated phenotypes. (DOCX) [file pgen.1003962.s008.docx]

**Table S1.** Mutant alleles used in this study and their associated phenotypes.

| **mutant allele** | **disrupts binding to:** | **major phenotypes:** | **references** |
| --- | --- | --- | --- |
| *tif32-Δ8* | reduced eIF3 binding to 40S by 40% | Slg-; severe Gcn-; reinitiation defect | [9] |
| *tif32-Box17* |  | Slg-; Gcn-; reinitiation defect | [10] |
| *tif32-Box6* |  | Slg-; Gcn-; reinitiation defect | [10] |
| *tif32-Box6+17* |  | severe Slg-; severe Gcn-; reinitiation defect | [10] |
| *prt1-W674A* | diminishes (by >90%) association of i/TIF34 and g/TIF35 with the rest of eIF3/MFC and 40S ribosomes | severe Ts-; Gcn- | [43] |
| *nip1-Box1* | reduced amounts of 40S-bound eIF3 and eIF5 *in vivo* | temperature-dependent Slg- | [S1-1] |
| *nip1- Δ60* | reduced amounts of 40S-bound eIF3 and eIF5 *in vivo* | Slg-; | [S1-1] |
| *tif34-Q258R* |  | Slg-; reduces the rate of scanning | [42] |
| *tif34-DD/KK* | separates eIF3 into the a-b-c and i-g modules; partially destabilizes 48S PICs | severe Ts-; produces a severe leaky scanning phenotype | [43] |
| *tif35-KLF* |  | Ts-; Gcn-; reinitiation defect; reduces processivity of scanning | [42] |
| *tif35-TKMQ* |  | Slg-; severe Gcn-; modest leaky scanning | L.C. and L.S.V. unpublished |
| *tif35-RLFT* |  | Slg-; severe Gcn- | L.C. and L.S.V. unpublished |
| *tif35-C121R* |  | Slg- | L.C. and L.S.V. unpublished |
| *hcr1 Δ* | destabilizes the MFC; reduces 40S binding of eIF3 | Slg-; reduces the levels of mature 40S ribosome; leaky scanning | [46]; [19]; [21] |
| *hcr1-NTA* | eliminates binding of HCR1 to eIF3 | leaky scanning | [21] |
| *hcr1-Box6* | eliminates binding of HCR1 to eIF3 |  | [22] |
| *hcr1-Box6+R/I* | eliminates binding of HCR1 to eIF3 | leaky scanning | [22] |
| *rli1-K116L* | eliminates binding to HCR1 in Y2H | lethal | [S1-2] |
| *rli1-K391L* | eliminates binding to HCR1 in Y2H | lethal | [S1-2] |
| *sup45-M48I* |  | Ts-; nonsense suppression; stop codon decoding defect | [29]; [30] |
| *sup45-Y410S* | decrease binding affinity to eRF3 | severe Ts-; nonsense suppression | [29]; [28] |
| *sup35-N536T* |  | Ts-; nonsense suppression | [29] |

slow growth (Slg^-^); temperature sensitivity (Ts^-^); prevents translational derepression of *GCN4* expression during amino acid starvation (Gcn^-^); multifactor complex (MFC); pre-initiation complex (PIC); yeast two-hybrid (Y2H)

9. Szamecz B, Rutkai E, Cuchalova L, Munzarova V, Herrmannova A, et al. (2008) eIF3a cooperates with sequences 5' of uORF1 to promote resumption of scanning by post-termination ribosomes for reinitiation on GCN4 mRNA. Genes Dev 22: 2414-2425.

10. Munzarová V, Pánek J, Gunišová S, Dányi I, Szamecz B, et al. (2011) Translation Reinitiation Relies on the Interaction between eIF3a/TIF32 and Progressively Folded cis-Acting mRNA Elements Preceding Short uORFs. PLoS Genet 7: e1002137.

43. Herrmannová A, Daujotyte D, Yang JC, Cuchalová L, Gorrec F, et al. (2012) Structural analysis of an eIF3 subcomplex reveals conserved interactions required for a stable and proper translation pre-Initiation complex assembly. Nucleic Acids Res 40: 2294-2311.

S1-1. Kouba T, Rutkai E, Karasková M, Valášek LS (2012) The eIF3c/NIP1 PCI domain interacts with RNA and RACK1/ASC1 and promotes assembly of the pre-initiation complexes. Nucleic Acids Research 40: 2683-2699.

42. Cuchalová L, Kouba T, Herrmannová A, Danyi I, Chiu W-l, et al. (2010) The RNA Recognition Motif of Eukaryotic Translation Initiation Factor 3g (eIF3g) Is Required for Resumption of Scanning of Posttermination Ribosomes for Reinitiation on GCN4 and Together with eIF3i Stimulates Linear Scanning. Mol Cell Biol 30: 4671-4686.

46. Valášek L, Hašek J, Trachsel H, Imre EM, Ruis H (1999) The *Saccharomyces cerevisiae HCRI* gene encoding a homologue of the p35 subunit of human translation eukaryotic initiation factor 3 (eIF3) is a high copy suppressor of a temperature-sensitive mutation in the Rpg1p subunit of yeast eIF3. J Biol Chem 274: 27567-27572.

19. Valášek L, Phan L, Schoenfeld LW, Valášková V, Hinnebusch AG (2001) Related eIF3 subunits TIF32 and HCR1 interact with an RNA recoginition motif in PRT1 required for eIF3 integrity and ribosome binding. EMBO J 20: 891-904.

21. ElAntak L, Wagner S, Herrmannová A, Karásková M, Rutkai E, et al. (2010) The indispensable N-terminal half of eIF3j co-operates with its structurally conserved binding partner eIF3b-RRM and eIF1A in stringent AUG selection. J Mol Biol 396: 1097-1116.

22. Chiu W-L, Wagner S, Herrmannová A, Burela L, Zhang F, et al. (2010) The C-Terminal Region of Eukaryotic Translation Initiation Factor 3a (eIF3a) Promotes mRNA Recruitment, Scanning, and, Together with eIF3j and the eIF3b RNA Recognition Motif, Selection of AUG Start Codons. Mol Cell Biol 30: 4415-4434.

S1-2. Kispal G, Sipos K, Lange H, Fekete Z, Bedekovics T, et al. (2005) Biogenesis of cytosolic ribosomes requires the essential iron-sulphur protein Rli1p and mitochondria. EMBO J 24: 589-598.

29. Bradley ME, Bagriantsev S, Vishveshwara N, Liebman SW (2003) Guanidine reduces stop codon read-through caused by missense mutations in SUP35 or SUP45. Yeast 20: 625-632.

30. Bertram G, Bell HA, Ritchie DW, Fullerton G, Stansfield I (2000) Terminating eukaryote translation: domain 1 of release factor eRF1 functions in stop codon recognition. Rna 6: 1236-1247.

28. Akhmaloka, Susilowati PE, Subandi, Madayanti F (2008) Mutation at tyrosine in AMLRY (GILRY like) motif of yeast eRF1 on nonsense codons suppression and binding affinity to eRF3. Int J Biol Sci 4: 87-95.
