# Supplementary material for: Translation Initiation Factors eIF3 and HCR1 Control Translation Termination and Stop Codon Read-Through in Yeast Cells
Source: PLoS Genet. 2013 Nov 21;9(11):e1003962. doi: 10.1371/journal.pgen.1003962 (PMC3836723; doi:10.1371/journal.pgen.1003962)
Supplement: Table S2 — Yeast strains used in this study. (DOCX) [file pgen.1003962.s009.docx]

**Table S2.** Yeast strains used in this study.

| **Strain** | **Genotype** | **Source or reference** |
| --- | --- | --- |
| del’32a9A^a^ | *MAT*a *leu2-3, -112 ura3-52 trp1Δ gcn2Δ a/tif32Δ* (sc *TIF32, URA3*) | [9] |
| YAH06^a^ | *MATa leu2-3,112 ura3-52 trp1**Δ prt1::hisG GCN2* (hc *PRT1 URA3*) | [21] |
| HMJ08^a^ | *MATa, trp1 leu2-3,-112 ura3-52 nip1Δ* (sc *NIP1-His URA3*) | [S2-1] |
| H450^a^ | *MAT*a *leu2-3,-112 ura3-52::GCN2 trp1Δ tif34Δ* (hc *TIF34 URA3)*) | [42] |
| H464^a^ | *MAT*a *leu2-3,-112 ura3-52::GCN2 trp1Δ tif35Δ* (hc *TIF35 URA3*) | [42] |
| PBH103 | *MATα ade1-14 trp1-289 his3-Δ200 leu2-3,112 ura3-52 sup35-N536T hcr1Δ::LEU2* | this study |
| PBH104 | *MATα ade1-14 trp1-289 his3-Δ200 leu2-3,112 ura3-52 sup45-M48I hcr1Δ::LEU2* | this study |
| PBH105 | *MATa ade1-14 trp1-289 his3-Δ200 leu2-3,112 ura3-52 sup45-Y410S hcr1Δ::LEU2* | this study |
| YLVH13^b^ | *MATα hcr1Δ::LEU2 ade2-1 trp1-1 can1-100 leu2-3 leu2-112 his3-11 his3-15 ura3* | [46] |
| L2334^c^ | *MAT*a *ade1-14* *trp1-289 his3-Δ200 leu2-3,112 ura3-52 sup35-N536T* | [29] |
| L2327^c^ | *MAT*a *ade1-14* *trp1-289 his3-Δ200 leu2-3,112 ura3-52 sup45-M48I* | [29] |
| L2521^c^ | *MAT*a *ade1-14* *trp1-289 his3-Δ200 leu2-3,112 ura3-52 sup45-Y410S* | [29] |
| 74D-694^c^ | *MAT*a *ade1-14* *trp1-289 his3-Δ200 leu2-3,112 ura3-52* | [S2-2] |
| PBH106 | *MATα trp1-289 his3-Δ200 leu2-3,112 ura3-52 sup45-Y410S a/tif32Δ* (sc *TIF32, URA3*) | this study |
| PBH107 | *MATα ade1-14* *trp1-289 his3-Δ200 leu2-3,112 ura3-52 sup35-N536T a/tif32Δ* (sc *TIF32, URA3*) | this study |
| del’32a9B^a^ | *MATα* *leu2-3, -112 ura3-52 trp1Δ gcn2Δ a/tif32Δ* (sc *TIF32, URA3*) | [20] |
| YDH353 | *MATa his3Δ1 leu2Δ0 ura3Δ0 met15Δ0 RLI1-MYC::HIS3* | [1] |
| H553^d^ | *MATa his3Δ1 leu2Δ0 met15Δ0 ura3Δ0 HCR1-TAP* | Thermo Scientific |
| H555^d^ | *MATa his3Δ1 leu2Δ0 met15Δ0 ura3Δ0 TIF32-TAP* | Thermo Scientific |
| H517^c^ | *MATa ade1-14 trp1-289 his3-Δ200 leu2-3,112 ura3-52 SUP35::TAP-HIS3* | this study |
| H3675^a^ | *MATa PRT1 leu2-3, 112 ura3-52 hcr1Δ* | [20] |
| H2879^a^ | *MATa PRT1 leu2-3, 112 ura3-52* | [S2-3] |
| W303 ^b^ | *MATa ade2 can1-100 his3-11 his3-15 leu2-3 leu2-112 trp1-1 ura3-1* | 1. Hopper |
| Tet-RLI1^b^ | *MATα ade2 can1-100 his3-11 his3-15 leu2-3 leu2-112 trp1-1 ura3-1 Tet::RLI1* | [S2-4] |
| CML476-SUP45 | *MATa ura3-52 leu2Δ1 his3Δ200 GAL2 CMVp(tetR’-SSN6)::LEU2 trp1::tTA* | [S2-5] |

^a-d^ Identical superscripts denote isogenic strain backgrounds.

9. Szamecz B, Rutkai E, Cuchalova L, Munzarova V, Herrmannova A, et al. (2008) eIF3a cooperates with sequences 5' of uORF1 to promote resumption of scanning by post-termination ribosomes for reinitiation on GCN4 mRNA. Genes Dev 22: 2414-2425.

21. ElAntak L, Wagner S, Herrmannová A, Karásková M, Rutkai E, et al. (2010) The indispensable N-terminal half of eIF3j co-operates with its structurally conserved binding partner eIF3b-RRM and eIF1A in stringent AUG selection. J Mol Biol 396: 1097-1116.

S2-1. Kouba T, Rutkai E, Karasková M, Valášek LS (2012) The eIF3c/NIP1 PCI domain interacts with RNA and RACK1/ASC1 and promotes assembly of the pre-initiation complexes. Nucleic Acids Research 40: 2683-2699.

42. Cuchalová L, Kouba T, Herrmannová A, Danyi I, Chiu W-l, et al. (2010) The RNA Recognition Motif of Eukaryotic Translation Initiation Factor 3g (eIF3g) Is Required for Resumption of Scanning of Posttermination Ribosomes for Reinitiation on GCN4 and Together with eIF3i Stimulates Linear Scanning. Mol Cell Biol 30: 4671-4686.

46. Valášek L, Hašek J, Trachsel H, Imre EM, Ruis H (1999) The *Saccharomyces cerevisiae HCRI* gene encoding a homologue of the p35 subunit of human translation eukaryotic initiation factor 3 (eIF3) is a high copy suppressor of a temperature-sensitive mutation in the Rpg1p subunit of yeast eIF3. J Biol Chem 274: 27567-27572.

29. Bradley ME, Bagriantsev S, Vishveshwara N, Liebman SW (2003) Guanidine reduces stop codon read-through caused by missense mutations in SUP35 or SUP45. Yeast 20: 625-632.

S2-2. Chernoff YO, Derkach IL, Inge-Vechtomov SG (1993) Multicopy SUP35 gene induces de-novo appearance of psi-like factors in the yeast Saccharomyces cerevisiae. Curr Genet 24: 268-270.

1. Dong J, Lai R, Nielsen K, Fekete CA, Qiu H, et al. (2004) The essential ATP-binding cassette protein RLI1 functions in translation by promoting preinitiation complex assembly. J Biol Chem 279: 42157-42168.

20. Nielsen KH, Valášek L, Sykes C, Jivotovskaya A, Hinnebusch AG (2006) Interaction of the RNP1 motif in PRT1 with HCR1 promotes 40S binding of eukaryotic initiation factor 3 in yeast. Mol Cell Biol 26: 2984-2998.

S2-3. Nielsen KH, Szamecz B, Valasek LJ, A., Shin BS, Hinnebusch AG (2004) Functions of eIF3 downstream of 48S assembly impact AUG recognition and GCN4 translational control. EMBO J 23: 1166-1177.

S2-4. Kispal G, Sipos K, Lange H, Fekete Z, Bedekovics T, et al. (2005) Biogenesis of cytosolic ribosomes requires the essential iron-sulphur protein Rli1p and mitochondria. EMBO J 24: 589-598.

S2-5. Yen K, Gitsham P, Wishart J, Oliver SG, Zhang N (2003) An improved tetO promoter replacement system for regulating the expression of yeast genes. Yeast 20: 1255-1262.
