# Supplementary material for: Translation Initiation Factors eIF3 and HCR1 Control Translation Termination and Stop Codon Read-Through in Yeast Cells
Source: PLoS Genet. 2013 Nov 21;9(11):e1003962. doi: 10.1371/journal.pgen.1003962 (PMC3836723; doi:10.1371/journal.pgen.1003962)
Supplement: Table S3 — Plasmids used in this study. (DOCX) [file pgen.1003962.s010.docx]

**Table S3.** Plasmids used in this study.

| **Plasmid** | **Description** | **Source of reference** |
| --- | --- | --- |
| pTH477 | high copy PGK-Renilla-Firefly R/T cassette (stop codon of Renilla is UGA-C; for read-through measurements) in *URA3* plasmid from YEplac195 | [14] |
| pTH460 | high copy PGK-Renilla-Firefly R/T cassette (stop codon of Renilla is replaced with CAA-C [coding triplet]; for control read-through measurements) in *URA3* plasmid from YEplac195 | [14] |
| YEp-R/T-UGAC-L | high copy PGK-Renilla-Firefly R/T cassette (stop codon of Renilla is UGA-C; for read-through measurements) in *LEU2* plasmid from YEplac181 | this study |
| YEp-R/T-CAAC-L | high copy PGK-Renilla-Firefly R/T cassette (stop codon of Renilla is replaced with CAA-C [coding triplet]; for control read-through measurements) in *LEU2* plasmid from YEplac181 | this study |
| YCp-a/TIF32-His-L | single copy wt *TIF32-His* in *LEU2* plasmid from YCplac111 | [10] |
| YCp-a/tif32-Box17-His | single copy *tif32-Box17-His* in *LEU2* plasmid from YCplac111 | [10 |
| YCp-a/tif32-Box6-His | single copy *tif32-Box6-His* in *LEU2* plasmid from YCplac111 | [10] |
| YCp-a/tif32-Box6+17-His | single copy *tif32-Box6+17-His* in *LEU2* plasmid from YCplac111 | [10] |
| YCp-a/tif32-Δ8-His-L | single copy *tif32-Δ8-His* in *LEU2* plasmid from YCplac111 | this study |
| pRS-b/PRT1-HisXS | low copy wt *PRT1* in *LEU2* plasmid from pRS315 | [21] |
| pRS-b/PRT1-W674A-His | low copy *prt1-W674A* in *LEU2* plasmid from pRS315 | [43] |
| YCpNIP1-Myc-L | single copy wt *NIP1-Myc* in *LEU2* plasmid from YCplac111 | [S3-1] |
| YCpNIP1-743A752(box1) | single copy *NIP1-Myc* containing 10 Ala substitutions between  amino acid residues 743 and 752, in *LEU2* plasmid from  YCplac111 | [S3-1] |
| YCpNIP1-∆60-MYC-L | single copy *NIP1-Myc* truncated by 60 amino acid residues, in *LEU2* plasmid from YCplac111 | [S3-1] |
| YCp-i/TIF34-HA | single copy wt *TIF34-HA* in *LEU2* plasmid from YCplac111 | [42 |
| \|  \| \| --- \|   YCpL-i/tif34-HA-3 (Q258R) | single copy *tif34-HA-Q258R* in *LEU2* plasmid from YCplac111 | [42] |
| YCp-i/TIF34-D207K-D224K-HA | single copy *TIF34-HA* containing D207K and D224K mutations in *LEU2* plasmid from YCplac111 | [43] |
| YCp22-g/TIF35-screen | single copy wt *TIF35-His* in *TRP1* plasmid from YCplac22 | [42] |
| YCp22-g/TIF35-KLF | single copy *TIF35-KLF-His* in*TRP1* plasmid from YCplac22 | [42] |
| YCp22-g/TIF35-TKMQ | single copy *TIF35-TKMQ-His* in *TRP1* plasmid from YCplac22 | this study |
| YCp22-g/TIF35-RLFT | single copy *TIF35-RLFT-His* in *TRP1* plasmid from YCplac22 | this study |
| YCp22-g/TIF35-C121R | single copy *TIF35-C121R-His* in *TRP1* plasmid from YCplac22 | this study |
| YEplac181 | high copy cloning vector, *LEU2* | [S3-2] |
| YEplac195 | high copy cloning vector, *URA3* | [S3-2] |
| YEpLVHCR1 | high copy wt *HCR1* in *LEU2* plasmid from YEplac181 | [46] |
| YEp-HCR1-DS-U | high copy *HCR1* coding region flanked by *Bam*HI and *Nco*I sites, respectively, in *URA* plasmid from YEplac195 | [21] |
| YEp-RLI1-L | high copy wt *RLI1* in *LEU2* plasmid from YEplac181 | this study |
| YEp-hcr1-NTD | high copy *hcr1-NTD* in *LEU2* plasmid from YEplac181 | [21] |
| YEp-hcr1-CTD | high copy *hcr1-CTD* in *LEU2* plasmid from YEplac181 | [21] |
| YEp-hcr1-NTA1 | high copy *hcr1-NTA1* in *LEU2* plasmid from YEplac181 | [21] |
| YEp-hcr1-box6 | high copy *hcr1-Box6* in *LEU2* plasmid from YEplac181 | [22] |
| YEp-hcr1-box6-R215I | high copy *hcr1-Box6-R215I*  in *LEU2* plasmid from YEplac181 | [22] |
| PDH177 | single copy wt *RLI1* in *URA3* plasmid from YCplac33 | [1] |
| YEp-rli1-K116L-L | high copy *rli1-K116L* in *LEU2* plasmid from YEplac181 | this study |
| YEp-RLI1-ndeI-L | high copy wt *RLI1* in *LEU2* plasmid from YEplac181 | this study |
| YEp-rli1-K391L-L | high copy *rli1-K391L* in *LEU2* plasmid from YEplac181 | this study |
| YEp-rli1-G224D,G225D-L | high copy *rli1-G224D,G225D* in *LEU2* plasmid from YEplac181 | this study |
| YEp-rli1-G470D,G471D-L | high copy *rli1-G470D,G471D* in *LEU2* plasmid from YEplac181 | this study |
| YEp-rli1-E493Q-L | high copy *rli1-E493Q* in *LEU2* plasmid from YEplac181 | this study |
| PDH184 | single copy P_GAL_-UBI-M-FH-rli1-G224D,G225D plasmid from YCplac111 | [1] |
| PDH185 | single copy P_GAL_-UBI-M-FH-rli1-G470D,G471D plasmid from YCplac111 | [1] |
| PDH202 | low copy *rli1-E493Q-myc_5_* plasmid from pRS315 | [1] |
| YEp-rli1-C25S-L | high copy *rli1-C25S* in *LEU2* plasmid from YEplac181 | this study |
| YEp-rli1-C61S-L | high copy *rli1-C61S* in *LEU2* plasmid from YEplac181 | this study |
| YEpSUI1-U | high copy *SUI1* in *LEU2* plasmid from YEplac181 | this study |
| pDSO166 | high copy *TIF11* in *LEU2* plasmid from YEplac181 | [S3-3] |
| pGEX-5X-3 | cloning vector for GST fusions | [S3-4] |
| pGEX- g/TIF34 | GST-g/Tif34 fusion plasmid from pGEX-4T-1 | [S3-5] |
| pGEX- g/TIF35 | GST-g/Tif35 fusion plasmid from pGEX-5X-3 | [S3-5] |
| pGEX-HCR1 | GST-HCR1 fusion plasmid from pGEX-5X-3 | [19] |
| pTH338 | T7 promoter plasmid containing a full length SUP45 gene | this study |
| pTH339 | T7 promoter plasmid containing the SUP45 N-domain | this study |
| pTH340 | T7 promoter plasmid containing the SUP45 N- and M-domains | this study |
| pTH341 | T7 promoter plasmid containing the SUP45 M- and C-domains | this study |
| pTH342 | T7 promoter plasmid containing the SUP45 C-domain | this study |
| TKB668 | high copy *YEF3* in *URA3* plasmid from YEPlac195 | a gift of T. Kinzy |
| pGEX-RLI1 | GST-RLI1 fusion plasmid from pGEX-5X-3 | this study |
| pGEX-SUP45 | GST-SUP45 fusion plasmid from pGEX-6P-1 | a gift of A. Hinnebusch |
| plig102-3 | Low copy *URA3* vector with *GCN4* leader point mutations containing uORF4 only at its original position in front of the *GCN4-lacZ* coding region | [S3-6] |
| YCplac22 | single copy cloning vector, *TRP1* | [S3-2] |
| YCp22-SUP45-W | single copy wt *SUP45* in *TRP1* plasmid from YCplac22 | this study |
| pGEX- g/tif35-NTD | GST-g/tif35-NTD fusion plasmid from pGEX-5X-3 | this study |
| pGEX-g/tif35-RRM | GST-g/tif35-RRM fusion plasmid from pGEX-5X-3 | this study |
| pTH422 | high copy wt *SUP45* in *URA3* | this study |
| pRS426-SUP35 | high copy wt *SUP35* in *URA3* | this study |
| pSP35-45 | high copy wt *SUP45* *SUP35* in *URA3* | [S3-7] |

14. Keeling KM, Lanier J, Du M, Salas-Marco J, Gao L, et al. (2004) Leaky termination at premature stop codons antagonizes nonsense-mediated mRNA decay in S. cerevisiae. RNA 10: 691-703.

10. Munzarová V, Pánek J, Gunišová S, Dányi I, Szamecz B, et al. (2011) Translation Reinitiation Relies on the Interaction between eIF3a/TIF32 and Progressively Folded cis-Acting mRNA Elements Preceding Short uORFs. PLoS Genet 7: e1002137.

21. ElAntak L, Wagner S, Herrmannová A, Karásková M, Rutkai E, et al. (2010) The indispensable N-terminal half of eIF3j co-operates with its structurally conserved binding partner eIF3b-RRM and eIF1A in stringent AUG selection. J Mol Biol 396: 1097-1116.

43. Herrmannová A, Daujotyte D, Yang JC, Cuchalová L, Gorrec F, et al. (2012) Structural analysis of an eIF3 subcomplex reveals conserved interactions required for a stable and proper translation pre-Initiation complex assembly. Nucleic Acids Res 40: 2294-2311.

S3-1. Kouba T, Rutkai E, Karasková M, Valášek LS (2012) The eIF3c/NIP1 PCI domain interacts with RNA and RACK1/ASC1 and promotes assembly of the pre-initiation complexes. Nucleic Acids Research 40: 2683-2699.

42. Cuchalová L, Kouba T, Herrmannová A, Danyi I, Chiu W-l, et al. (2010) The RNA Recognition Motif of Eukaryotic Translation Initiation Factor 3g (eIF3g) Is Required for Resumption of Scanning of Posttermination Ribosomes for Reinitiation on GCN4 and Together with eIF3i Stimulates Linear Scanning. Mol Cell Biol 30: 4671-4686.

S3-2. Gietz RD, Sugino A (1988) New yeast-Escherichia coli shuttle vectors constructed with in vitro mutagenized yeast genes lacking six-base pair restriction sites. Gene 74: 527-534.

46. Valášek L, Hašek J, Trachsel H, Imre EM, Ruis H (1999) The *Saccharomyces cerevisiae HCRI* gene encoding a homologue of the p35 subunit of human translation eukaryotic initiation factor 3 (eIF3) is a high copy suppressor of a temperature-sensitive mutation in the Rpg1p subunit of yeast eIF3. J Biol Chem 274: 27567-27572.

22. Chiu W-L, Wagner S, Herrmannová A, Burela L, Zhang F, et al. (2010) The C-Terminal Region of Eukaryotic Translation Initiation Factor 3a (eIF3a) Promotes mRNA Recruitment, Scanning, and, Together with eIF3j and the eIF3b RNA Recognition Motif, Selection of AUG Start Codons. Mol Cell Biol 30: 4415-4434.

1. Dong J, Lai R, Nielsen K, Fekete CA, Qiu H, et al. (2004) The essential ATP-binding cassette protein RLI1 functions in translation by promoting preinitiation complex assembly. J Biol Chem 279: 42157-42168.

S3-3. Olsen DS, Savner EM, Mathew A, Zhang F, Krishnamoorthy T, et al. (2003) Domains of eIF1A that mediate binding to eIF2, eIF3 and eIF5B and promote ternary complex recruitment *in vivo*. EMBO J 22: 193-204.

S3-4. Smith DB, Johnson KS (1988) Single-step purification of polypeptides expressed in Escherichia coli as fusions with glutathione S-transferase. Gene 67: 31-40.

S3-5. Asano K, Phan L, Anderson J, Hinnebusch AG (1998) Complex formation by all five homologues of mammalian translation initiation factor 3 subunits from yeast *Saccharomyces cerevisiae*. J Biol Chem 273: 18573-18585.

19. Valášek L, Phan L, Schoenfeld LW, Valášková V, Hinnebusch AG (2001) Related eIF3 subunits TIF32 and HCR1 interact with an RNA recoginition motif in PRT1 required for eIF3 integrity and ribosome binding. EMBO J 20: 891-904.

S3-6. Grant CM, Miller PF, Hinnebusch AG (1994) Requirements for intercistronic distance and level of eIF-2 activity in reinitiation on GCN4 mRNA varies with the downstream cistron. Mol Cell Biol 14: 2616-2628.

S3-7. Bidou L, Stahl G, Hatin I, Namy O, Rousset JP, et al. (2000) Nonsense-mediated decay mutants do not affect programmed -1 frameshifting. Rna 6: 952-961.
