# Supplementary material for: Translation Initiation Factors eIF3 and HCR1 Control Translation Termination and Stop Codon Read-Through in Yeast Cells
Source: PLoS Genet. 2013 Nov 21;9(11):e1003962. doi: 10.1371/journal.pgen.1003962 (PMC3836723; doi:10.1371/journal.pgen.1003962)
Supplement: Table S4 — Primers used in this study. (DOCX) [file pgen.1003962.s011.docx]

**Table S4.** Primers used in this study.

| **Primer name** | **Primer sequence (5´to 3´)** |
| --- | --- |
| SG‑TIF32D8bamHI | GCGGATCCATATGAGATTAGCTGAAATG |
| BS-TIF32D8nheI-R | GGGGCTAGCGGGTCCCTGCCCCCCTTGGCCAATC |
| TIF35 NdeI | TAACGACCATATGACCATGAA |
| MM2r 3gTLKVr | ACACATATCATCACGTTCTCT |
| y3gTKMQ (RNP2) | GACTCTAGAGAACGTGATGATATGTGTgctTTGgcaATTgctgcaGTTAATGAAAATGCCGATGAAAATAGT |
| y3g XhoIr | GTGCATCTCGAGCTAATGATG |
| MM1r | TGATTTACCTGTTTCTTTGT |
| y3gRLFT (RNP1) | GTTAGAAACAAAGAAACAGGTAAATCAgcaGGTgcaGCCgcaGTTgcaTTTTCGAGCGAAGAAGTTGCCGAACAA |
| PB-RLI1ndeI | AATAACATATGAGTGATAAAAACAGTCGT |
| PB-RLI1-K116LaccI-R | AATAAGTAGACAGACCAATACCGTTGGTACCGAC |
| PB-RLI1accI-R | CAAGGCGGTAGACTTAC |
| PB-RLI1ncoI | GCAAATACCATGGATAG |
| PB-RLI1ndeI-R | ACGACTGTTTTTATCACTCATATGGGTCTGTCGTGTTTTCTTAAG |
| PB-RLI1-K391L-R | AGACCGGTACCGTTTTCACCCAT |
| PB-RLI1-K391L | ATGGGTGAAAACGGTACCGGTCTGACCACTTTGATCAAATTACTA |
| PB-RLI1termXbaI-R | GGCGTATATCTAGAAATAAACAACC |
| PB-RLI1xbaI-R | AATAATCTAGATTAAATACCGGTGTTATCCAA |
| PB-RLI1-C25S | CTAAACGTTCGTGTCCCG |
| PB-RLI1-C25S-R | CGGGACACGAACGTTTAGACTCTTGACGACACTTTTTTGG |
| PB-RLI1-C61S | CCGTTAAGAAATGTCCATTTG |
| PB-RLI1-C61S-R | CAAATGGACATTTCTTAACGGAAATACCACAACCAATAC |
| Sup45_D1f | CGGGCCATGGAGATGGATAACGAGGTTGAAAAAAA |
| Sup45_D3r | CGGGCCTGCAGTTAAATGAAATCATAGTCGGAT |
| Sup45_D1r | CGGGCCTGCAGTTATTGAAGCAATTCCGAAAGAAC |
| Sup45_D2r | CGGGCCTGCAGTTAATTGGCCAACGCTTCGGC |
| Sup45_D2f | CGGGCCATGGAGGCTGACGACAAGTTCGGT |
| Sup45_D3r | CGGGCCTGCAGTTAAATGAAATCATAGTCGGAT |
| Sup45_D3f | CGGGCCATGGAGGTCAAGTATGTTCAAGAAAAGA |
| PB-RLI1smaI | AATAACCCGGGTCATGAGTGATAAAAACAGTCGT |
| PB-RLI1xhoI-R | AATAACTCGAGTTAAATACCGGTGTTATCCAA |
| PB-SUP45SphI | AATAAGCATGCGCTACATCATTTCCCCCAATAGC |
| PB-SUP45SacI-R | AATAAGAGCTCCGAGGCTTTTGAAGAGAAACTCTCC |
| pGEX35NTD | AATAAGGATCCCCATGAGTGAAGTTGCACCAGAG |
| pGEX35NTDr | AATAACTCGAGctaAGAGTCTCTGTAAGCATCGGA |
| pGEX35RRM | AATAAGGATCCCCAGAGAACGTGATGATATGTGT |
| pGEX35RRMr | AATAACTCGAGCTATTCCTTAACCTTAGGTTTGGA |
