# Supplementary material for: Translation Initiation Factors eIF3 and HCR1 Control Translation Termination and Stop Codon Read-Through in Yeast Cells
Source: PLoS Genet. 2013 Nov 21;9(11):e1003962. doi: 10.1371/journal.pgen.1003962 (PMC3836723; doi:10.1371/journal.pgen.1003962)
Supplement: Text S1 — Supporting Materials and Methods. (DOCX) [file pgen.1003962.s012.docx]

**Supplementary Information**

**Material and Methods**

***Construction of yeast strains and plasmids***

List of all strains used throughout this study can be found in Table S2.

To generate *TIF32^+^*, *tif32^Box17^, tif32^Box6^*, *tif32^Box6+17^* and *tif32^∆8^* mutant strains (all showing in Figure 1), del’32a9A was transformed with YCp-a/TIF32-His-L, YCp-a/tif32-Box17-His, YCp-a/tif32-Box6-His , YCp-a/tif32-Box6+17-His, and YCp-a/tif32-Δ8-His-L, respectively, and the resident *URA3*-based plasmid carrying wt *TIF32* was evicted on 5-FOA-containing medium.

To generate *PRT1^+^* and *prt1^W647A^* mutant strains (Figure 1), YAH06 was transformed with pRS-b/PRT1-HisXS and pRS-b/PRT1-W674A-His, respectively, and the resident *URA3*-based plasmid carrying wt *PRT1* was evicted on 5-FOA-containing medium.

To generate *NIP1^+^*, *nip1^Box1^* and *nip1^∆60^* mutant strains (Figure 1), HMJ08 was transformed with YCpNIP1-Myc-L, YCpNIP1-743A752(box1) and YCpNIP1-∆60-MYC-L, respectively, and the resident *URA3*-based plasmid carrying wt *NIP1* was evicted on 5-FOA-containing medium.

To generate *TIF34*^+^, *tif34^Q258R^* and *tif34^DD/KK^* mutant strains (Figure 1), H450 was transformed with YCp-i/TIF34-HA, YCpL-i/tif34-HA-3 (Q258R) and YCp-i/TIF34-D207K-D224K-HA, respectively, and the resident *URA3*-based plasmid carrying wt *TIF34* was evicted on 5-FOA-containing medium.

To generate *TIF35^+^*, *tif35^KLF^*, *tif35^TKMQ^*, *tif35^RLFT^* and *tif35^C121R^* mutant strains (Figure 1), H464 was transformed with YCp22-g/TIF35-screen, YCp22-g/tif35-KLF, YCp22-g/tif35-TKMQ, YCp22-g/tif35-RLFT and YCp22-g/tif35-C121R, respectively, and the resident *URA3*-based plasmid carrying wt *TIF35* was evicted on 5-FOA-containing medium.

Strains PBH103 [*hcr1Δ sup35^N536T^*], PBH104 [*hcr1Δ sup45^M48I^*], and PBH105 [*hcr1Δ sup45^Y410S^*] were all generated by a genetic cross of YLVH13 with L2334, L2337, and L2521, respectively, selecting for a haploid ascospore showing the Ts^-^ phenotype and being prototrophic for leucine.

PBH106 [*tif32Δ sup45^Y410S^*] and PBH107 [*tif32Δ sup35^N536T^*] were generated by a genetic cross of del’32a9B with L2521 and L2334, respectively, selecting for a haploid ascospore showing the Ts^-^ phenotype and being unable to lose the resident *URA3*-based cover plasmid.

TAP-tagging at the chromosomal locus of SUP35 was performed in strain 74D-694 as described [1] producing H517.

List of all plasmids and PCR primers used throughout this study can be found in Tables S3 and S4, respectively.

YEp-R/T-UGAC-L and YEp-R/T-CAAC-L were constructed by inserting the 4567-bp *Alw*NI-*Nsi*I fragment from pTH477 and pTH460, respectively, into YEplac181 digested by AlwNI-NsiI.

YCp-a/tif32-Δ8-His-L was made by inserting the *Bam*HI-*Xba*I digested PCR product obtained with primers SG‑TIF32D8bamHI and BS-TIF32D8nheI-R using pRS-eIF3a-Δ8-His-L as a template into *Bam*HI-*Xba*I digested YCp-a/TIF32-His-L.

YCp22-g/TIF35-TKMQ was generated by fusion PCR. The following pairs of primers were used for separate PCR amplifications using YCp22-g/TIF35-screen as template: (1) TIF35 NdeI – MM2r 3gTLKVr; and (2) y3gTKMQ (RNP2) – y3g XhoIr. The PCR products thus obtained were used in a 1:1 ratio as templates for the third PCR amplification using primers y3g XhoIr and TIF35 NdeI. The resulting PCR product was digested with *Nde*I and *Xho*I and ligated with *Nde*I*-*XhoI-cleaved YCp22-g/TIF35-screen.

YCp22-g/TIF35-RLFT was generated by fusion PCR. The following pairs of primers were used for separate PCR amplifications using YCp22-g/TIF35-screen as template: (1) TIF35 NdeI – MM1r; and (2) y3gRLFT (RNP1) – y3g XhoIr. The PCR products thus obtained were used in a 1:1 ratio as templates for the third PCR amplification using primers y3g XhoIr and TIF35 NdeI. The resulting PCR product was digested with *Nde*I and *Xho*I and ligated with *Nde*I*-*XhoI-cleaved YCp22-g/TIF35-screen.

YCp22-g/TIF35-C121R was obtained by random mutagenesis of the template plasmid YCp22-g/TIF35-screen with help of XL1-Red Competent Cells (Stratagene).

YEp-RLI1-L was constructed by inserting the 2887-bp *Spe*I-*Pst*I fragment from PDH177 into YEplac181 digested with *Xba*I-*Pst*I.

YEp-rli1-K116L-L was made by inserting the *Nde*I-*Acc*I digested PCR product obtained with primers PB-RLI1ndeI and PB-RLI1-K116LaccI-R using YEp-RLI1-L as the template into *Nde*I-*Acc*I digested YEp-RLI1-ndeI-L. To create YEp-RLI1-ndeI-L, fusion PCR was employed. The following pairs of primers were used for separate PCR amplifications using YEp-RLI1-L as template: (1) PB-RLI1ndeI – PB-RLI1accI-R; and (2) PB-RLI1ncoI – PB-RLI1ndeI-R. The PCR products thus obtained were used in a 1:1 ratio as templates for the third PCR amplification using primers PB-RLI1ncoI and PB-RLI1accI-R. The resulting PCR product was digested with *Nco*I and *Acc*I and ligated with *Nco*I-*Acc*I-cleaved YEp-RLI1-L.

YEp-rli1-K391L-L was generated by fusion PCR. The following pairs of primers were used for separate PCR amplifications using YEp-RLI1-ndeI-L as template: (1) PB-RLI1ndeI – PB-RLI1-K391L-R; and (2) PB-RLI1-K391L – PB-RLI1termXbaI-R. The PCR products thus obtained were used in a 1:1 ratio as templates for the third PCR amplification using primers PB-RLI1ndeI and PB-RLI1termXbaI-R. The resulting PCR product was digested with *Nde*I and *Xba*I and ligated with *Nde*I-*Xba*I-cleaved YEp-RLI1-ndeI-L.

YEp-rli1-G224D,G225D-L, YEp-rli1-G470D,G471D-L, and YEp-rli1-E493Q-L were made by inserting the *Nde*I-*Xba*I digested PCR product obtained with primers PB-RLI1ndeI and PB-RLI1xbaI-R using PDH184, PDH185, and PDH202 as templates, respectively, into *Nde*I-*Xba*I digested YEp-RLI1-ndeI-L.

YEp-rli1-C25S-L was generated by fusion PCR. The following pairs of primers were used for separate PCR amplifications using YEp-RLI1-ndeI-L as template: (1) PB-RLI1ndeI – PB-RLI1-C25S-R; and (2) PB-RLI1-C25S – PB-RLI1accI-R. The PCR products thus obtained were used in a 1:1 ratio as templates for the third PCR amplification using primers PB-RLI1ndeI and PB-RLI1accI-R. The resulting PCR product was digested with *Nde*I and *Acc*I and ligated with *Nde*I-*Acc*I-cleaved YEp-RLI1-ndeI-L.

YEp-rli1-C61S-L was generated by fusion PCR. The following pairs of primers were used for separate PCR amplifications using YEp-RLI1-ndeI-L as template: (1) PB-RLI1ndeI – PB-RLI1-C61S-R; and (2) PB-RLI1-C61S – PB-RLI1accI-R. The PCR products thus obtained were used in a 1:1 ratio as templates for the third PCR amplification using primers PB-RLI1ndeI and PB-RLI1accI-R. The resulting PCR product was digested with *Nde*I and *Acc*I and ligated with *Nde*I-*Acc*I-cleaved YEp-RLI1-ndeI-L.

To generate YEpSUI1-L, a *Sac*I-*Hind*III fragment from YEpSUI1-U [2] was ligated into *Sac*I-*Hind*III-cleaved YEplac181 [3].

Plasmids used for the generation of *in vitro*-translated eRF1 fragments (pTH339-342) were generated as follows. Fragments of the *SUP45* gene were generated by PCR using yeast genomic DNA as template. The primers used were Sup45_D1f and Sup45_D3r (for eRF1(fl)); Sup45_D1f and Sup45_D1r (for eRF1-N); Sup45_D1f and Sup45_D2r (for eRF1-NM); Sup45_d2f and Sup45_D3r (for eRF1-MC); and Sup45_D3f and Sup45_D3r (for eRF1-C). All primers added *Nco*I and *Not*I sites at the 5’- and 3’-ends of the PCR products, respectively, and also introduced translation start- and stop codons where required. The DNA fragments were then cloned into the yeast two-hybrid binding domain vector pGBK-T7 (Clontech Europe, France), which also contains a T7 transcription start site upstream of the gene’s start codon, using *NcoI* and *NotI* sites.

pGEX-RLI1 expression plasmid was constructed by insertion of the corresponding *Sma*I-*Xho*I-digested PCR product amplified from YEp-RLI1-L using the primers PB-RLI1smaI and PB-RLI1xhoI-R into *Sma*I-*Xho*I-digested pGEX-5X-3 [3].

pGEX- g/tif35-NTD expression plasmid was constructed by insertion of the corresponding *Bam*HI-*Xho*I-digested PCR product amplified from YCp22-g/TIF35-screen using the primers pGEX35NTD and pGEX35NTDr into *Bam*HI-*Xho*I-digested pGEX-5X-3 [3].

pGEX- g/tif35-RRM expression plasmid was constructed by insertion of the corresponding *Bam*HI-*Xho*I-digested PCR product amplified from YCp22-g/TIF35-screen using the primers pGEX35RRM and pGEX35RRMr into *Bam*HI-*Xho*I-digested pGEX-5X-3 [3].

YCp22-SUP45-W was made by inserting the *Sph*I-*Sac*I-digested PCR product (2033 bp-long) obtained from genomic DNA using primers PB-SUP45SphI and PB-SUP45SacI-R into the *Sph*I-*Sac*I-cut YCplac22.

**References**

1. Puig O, Caspary F, Rigaut G, Rutz B, Bouveret E, et al. (2001) The Tandem Affinity Purification (TAP) method: a general procedure of protein complex purification. Methods 24: 218-229.

2. Valášek L, Nielsen KH, Zhang F, Fekete CA, Hinnebusch AG (2004) Interactions of Eukaryotic Translation Initiation Factor 3 (eIF3) Subunit NIP1/c with eIF1 and eIF5 Promote Preinitiation Complex Assembly and Regulate Start Codon Selection. Mol Cell Biol 24: 9437-9455.

3. Gietz RD, Sugino A (1988) New yeast-Escherichia coli shuttle vectors constructed with in vitro mutagenized yeast genes lacking six-base pair restriction sites. Gene 74: 527-534.
